# Metabolic Network for the Biosynthesis of Intra- and Extracellular α-Glucans Required for Virulence of *Mycobacterium tuberculosis*

**DOI:** 10.1371/journal.ppat.1005768

**Published:** 2016-08-11

**Authors:** Hendrik Koliwer-Brandl, Karl Syson, Robert van de Weerd, Govind Chandra, Ben Appelmelk, Marina Alber, Thomas R. Ioerger, William R. Jacobs, Jeroen Geurtsen, Stephen Bornemann, Rainer Kalscheuer

**Affiliations:** 1 Institute for Medical Microbiology and Hospital Hygiene, Heinrich-Heine-University Düsseldorf, Düsseldorf, Germany; 2 Department of Biological Chemistry, John Innes Centre, Norwich, United Kingdom; 3 Department of Medical Microbiology and Infection Control, VU University Medical Center, Amsterdam, The Netherlands; 4 Department of Molecular Microbiology, John Innes Centre, Norwich, United Kingdom; 5 Department of Computer Science and Engineering, Texas A&M University, College Station, Texas, United States of America; 6 Howard Hughes Medical Institute, Department of Microbiology and Immunology, Albert Einstein College of Medicine, Bronx, New York, United States of America; 7 Institute for Pharmaceutical Biology and Biotechnology, Heinrich-Heine-University Düsseldorf, Düsseldorf, Germany; National Institutes of Health, UNITED STATES

## Abstract

*Mycobacterium tuberculosis* synthesizes intra- and extracellular α-glucans that were believed to originate from separate pathways. The extracellular glucose polymer is the main constituent of the mycobacterial capsule that is thought to be involved in immune evasion and virulence. However, the role of the α-glucan capsule in pathogenesis has remained enigmatic due to an incomplete understanding of α-glucan biosynthetic pathways preventing the generation of capsule-deficient mutants. Three separate and potentially redundant pathways had been implicated in α-glucan biosynthesis in mycobacteria: the GlgC-GlgA, the Rv3032 and the TreS-Pep2-GlgE pathways. We now show that α-glucan in mycobacteria is exclusively assembled intracellularly utilizing the building block α-maltose-1-phosphate as the substrate for the maltosyltransferase GlgE, with subsequent branching of the polymer by the branching enzyme GlgB. Some α-glucan is exported to form the α-glucan capsule. There is an unexpected convergence of the TreS-Pep2 and GlgC-GlgA pathways that both generate α-maltose-1-phosphate. While the TreS-Pep2 route from trehalose was already known, we have now established that GlgA forms this phosphosugar from ADP-glucose and glucose 1-phosphate 1000-fold more efficiently than its hitherto described glycogen synthase activity. The two routes are connected by the common precursor ADP-glucose, allowing compensatory flux from one route to the other. Having elucidated this unexpected configuration of the metabolic pathways underlying α-glucan biosynthesis in mycobacteria, an *M*. *tuberculosis* double mutant devoid of α-glucan could be constructed, showing a direct link between the GlgE pathway, α-glucan biosynthesis and virulence in a mouse infection model.

## Author Summary

Capsule formation is critical for the virulence of many bacterial and fungal pathogens. *Mycobacterium tuberculosis* cells are known to be surrounded by a capsule layer that is mainly composed of an α-glucan glucose polymer that resembles glycogen. Progress in understanding its role in the virulence of this important human pathogen has been held back by a lack of knowledge of its biosynthesis, preventing the generation of α-glucan-deficient mutants that could be tested in animal infection models. In this work, we unraveled an unexpected metabolic network configuration revealing the exclusive production of both intracellular and capsular α-glucans by the maltosyltransferase GlgE in mycobacteria. GlgE polymerizes an α-maltose 1-phosphate building block, which is generated by two alternative pathways that are connected by a common intermediate allowing rechanneling of flux from one route to the other. Elucidation of this unexpected configuration of the metabolic pathways underlying α-glucan biosynthesis allowed the rational construction of an *M*. *tuberculosis* mutant strain devoid of α-glucan, showing a direct link between the GlgE pathway, α-glucan biosynthesis and virulence in a mouse infection model.

## Introduction

More than 100 years after its discovery by Robert Koch, *Mycobacterium tuberculosis*, the etiologic agent of tuberculosis, still remains an unresolved global public health threat. New and more effective chemotherapies for the treatment of tuberculosis are urgently required. Findings in recent years suggest that α-glucan biosynthesis in *M*. *tuberculosis* may be an attractive process that offers several vulnerable steps that could be exploitable in the development of novel treatment options. Many bacteria produce α-1,4/α-1,6-glucan. However, while this glucose polymer is typically assembled intracellularly in a glycogen-like storage form, *M*. *tuberculosis* also deposits α-glucan extracellularly as a major component of the capsule [1, 2]. Anecdotal reports have suggested the presence of a capsular layer surrounding mycobacteria for a long time [3–5], but only recently has this layer been visualized in a near native state [6]. *In vitro* experiments using purified capsular α-glucan demonstrated that it can interact with complement receptor 3, thus mediating binding of *M*. *tuberculosis* to phagocytic cells [1, 7]. Capsular α-glucan also blocks dendritic cell functions [8] and interacts with the C-type lectin receptor DC-SIGN [9]. Furthermore, consistent with the general importance of the capsule for virulence of many bacterial and fungal pathogens, an *M*. *tuberculosis* mutant with a somewhat reduced amount of capsular α-glucan showed impaired virulence [10]. Collectively, these findings indicate that capsular α-glucans may be important for *M*. *tuberculosis* pathogenesis by interacting with mammalian host cells and influencing the immune response to *M*. *tuberculosis*. However, its precise role in virulence is unclear because the genes involved in its biosynthesis and export have not been conclusively elucidated, precluding the generation of capsule-deficient mutants.

The properties of intracellular (i.e. cytosolic) α-glucan (often referred to as glycogen) and extracellular (i.e. capsular) α-glucan from mycobacteria are very similar [10–13], suggesting a common biosynthetic origin. Most bacterial species are believed to employ the classical GlgC-GlgA pathway for the formation of glycogen-like α-glucans [14]. The first step in this pathway is the formation of ADP-glucose from glucose 1-phosphate by the ADP-glucose pyrophosphorylase GlgC. Subsequently, ADP-glucose is polymerized to give linear α-1,4-glucans by the glycogen synthase GlgA. Finally, the branching enzyme GlgB introduces α-1,6-branches. However, in contrast to this general scheme, α-glucan biosynthesis in *M*. *tuberculosis* exhibits increased complexity because two additional pathways appear to be involved (S1 Fig). In the first alternative pathway, the glucosyltransferase Rv3032 is thought to synthesize linear α-1,4-glucan by utilizing both UDP-glucose and ADP-glucose as activated donor substrates. This glycosyltransferase has been reported to contribute to the production of higher-molecular weight α-glucans in *M*. *tuberculosis*. However, its main role appears to be the formation of specialized oligomeric glucan derivatives, known as methylglucose lipopolysaccharides (MGLPs) [15]. The second alternative pathway is the recently discovered TreS-Pep2-GlgE pathway, which converts the abundant disaccharide trehalose into glucan polymers in four steps [16]. First, trehalose is isomerized to maltose by trehalose synthase TreS [17–19] with subsequent phosphorylation by maltokinase Pep2 to give maltose 1-phosphate (M1P) [19–21]. The phosphosugar M1P serves as the substrate for the key enzyme of this pathway, the maltosyltransferase GlgE, which transfers maltosyl units from the donor substrate M1P to the non-reducing end of α-glucan acceptors molecules, leading to linear α-1,4-chain elongation [22, 23]. Finally, the linear α-1,4-glucans are branched with α-1,6-linkages by the branching enzyme GlgB [13, 16].

The co-existence of three possible routes for synthesizing α-glucans in mycobacteria is unprecedented and raises the questions whether they are functionally redundant in the biosynthesis of α-glucan, or whether each is dedicated to the production of either intracellular glycogen-like material, capsular α-glucan or MGLPs. In this study, we therefore investigated how these three alternative pathways contribute to the production of intracellular and capsular α-glucans in mycobacteria. Employing comprehensive genetic manipulation of mycobacteria combined with metabolic analyses of mutant strains and enzyme characterization, we revealed an intricate and unexpected convergence of the TreS-Pep2-GlgE and the GlgC-GlgA pathways in α-glucan biosynthesis as they both produce the M1P building block required for α-glucan production by the key enzyme GlgE. Unravelling the configuration of the metabolic pathways underlying α-glucan biosynthesis enabled the generation of α-glucan-deficient mutant strains for the first time, allowing the study of the link between the GlgE pathway, the formation of α-glucan and the virulence of *M*. *tuberculosis* in an animal model.

## Results

### An alternative route for M1P formation in *Mycobacterium smegmatis*

**Fig 1:**
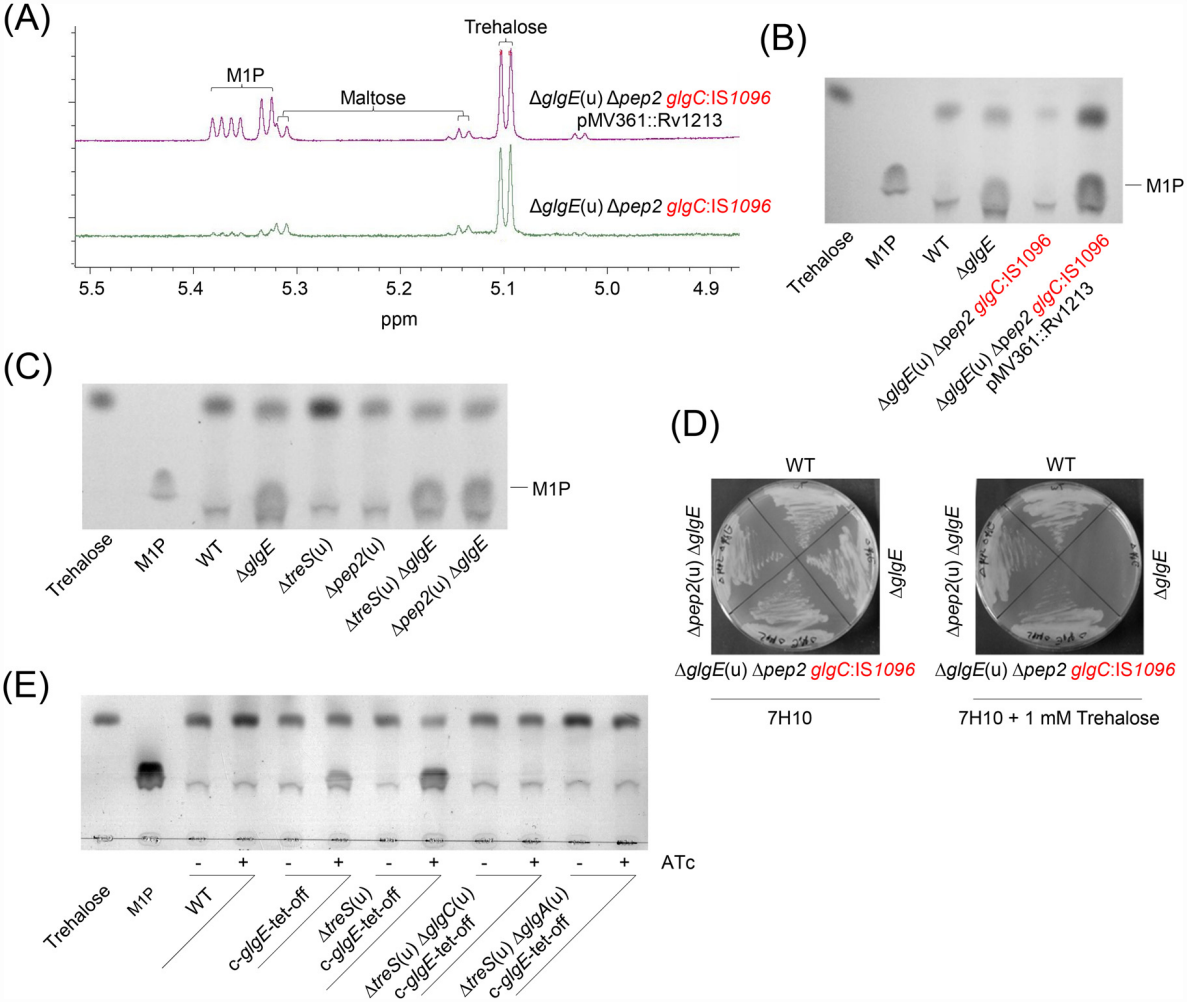
Evidence for an alternative route to M1P in *M*. *smegmatis* independent of TreS-Pep2 and trehalose. (A) Heterologous expression of the *M*. *tuberculosis glgC* gene (Rv1213) restores M1P accumulation in the *M*. *smegmatis* Δ*glgE* Δ*pep2* double mutant harboring a spontaneous IS*1096* element insertion in the endogenous *glgC* locus (i.e. *M*. *smegmatis* Δ*glgE* Δ*pep2 glgC*:IS*1096*). Equivalent quantities of crude extracts of *M*. *smegmatis* strains were analyzed using ^1^H NMR spectroscopy. The assignment of the peaks was based on our previous studies [16, 18]. (B) Hot water extracts from 1 ml culture aliquots of *M*. *smegmatis* strains (normalized to OD_600 nm_ = 0.5) were analyzed by TLC, demonstrating M1P accumulation in the *M*. *smegmatis* Δ*glgE* Δ*pep2 glgC*:IS*1096* strain expressing the *M*. *tuberculosis glgC* gene (Rv1213). M1P and trehalose (5 μg each) were used as standards. (C) *M*. *smegmatis* Δ*pep2*(u) Δ*glgE* and Δ*treS*(u) Δ*glgE* double mutants accumulate M1P. TLC analysis was performed as described in (B). (D) The *M*. *smegmatis* Δ*pep2*(u) Δ*glgE* mutant is trehalose resistant despite accumulating M1P, indicating trehalose-independent M1P formation. Strains were grown on Middlebrook 7H10 agar plates with or without 1 mM trehalose and incubated at 37°C for 3 days. (E) Conditional silencing of the *glgE* gene in *M*. *smegmatis* mutant strains reveals the requirement of GlgC and GlgA for the alternative route to M1P synthesis. Cells of the indicated conditional c-*glgE*-tet-off mutant strains were cultivated for 24 h with or without 1 μg ml^-1^ ATc as indicated, and hot water extracts from 1 ml culture aliquots (normalized to OD_600 nm_ = 0.5) were analyzed by TLC.

The maltosyltransferase GlgE (systematic name α-maltose 1-phosphate:(1→4)-α-D-glucan 4-α-D-maltosyltransferase) is unusual in that a phosphosugar, M1P, serves as the activated donor substrate. By contrast, most known glycosyltransferases rely on nucleotide-bound sugars as donor substrates. The only pathway known so far for the synthesis of M1P uses the abundant intracellular sugar trehalose (α,α-1,1-diglucose), which is isomerized to maltose (α-1,4-diglucose) by trehalose synthase TreS [17–19], followed by ATP-driven phosphorylation of maltose to M1P catalyzed by maltokinase Pep2 [19–21]. Consequently, M1P formation has been reported to be prevented by blocking TreS or Pep2, e.g. in *M*. *smegmatis* Δ*glgE*(u) Δ*treS* or Δ*glgE*(u) Δ*pep2* double mutants [16]. These data suggested the only route to M1P in *M*. *smegmatis* and other mycobacteria is through the TreS-Pep2 pathway from trehalose [16]. We have now analyzed extracts of the *M*. *smegmatis* Δ*glgE*(u) Δ*pep2* double mutant using a more sensitive method (^1^H-NMR spectroscopy instead of thin-layer chromatography, TLC), and were surprised to detect small amounts of M1P, despite the lack of Pep2 (Fig 1A). Mass spectrometry also confirmed the presence of an ion consistent with the presence of M1P.

Next, DNA microarray analyses were performed in order to find clues to explain the formation of M1P. We observed that the *glgC* gene in the *M*. *smegmatis* Δ*glgE*(u) Δ*pep2* mutant was downregulated compared with the parental strain control (4.83-fold), suggesting a stable, inadvertent spontaneous mutation of this locus. We therefore PCR-amplified the *glgC* locus and sequenced it. This revealed an intrachromosomal IS element mobilization had occurred in this strain with insertion of IS*1096* 10 bp upstream of the ATG start codon of the *glgC* gene, which likely impaired gene expression. This means that the *M*. *smegmatis* strain *de facto* represented a Δ*glgE*(u) Δ*pep2 glgC*:IS*1096* triple mutant. It is likely that the observed IS element mobilization was a result of M1P toxicity caused by *glgE* gene deletion, maybe providing a mechanism to suppress M1P stress. We thus complemented the defective *glgC* gene in the *M*. *smegmatis* Δ*glgE*(u) Δ*pep2 glgC*:IS*1096* mutant by heterologous expression of the *M*. *tuberculosis glgC* gene (Rv1213). To our surprise, we found that this “repair” of the *glgC* locus strongly enhanced M1P accumulation, as revealed by ^1^H-NMR (Fig 1A) and TLC analyses (Fig 1B), even though M1P synthesis by Pep2 was not possible. Next, we corroborated this result by re-generating *M*. *smegmatis* double mutants by first deleting either the *treS* or *pep2* gene, followed by deletion of the *glgE* gene, in order to minimize any potential M1P stress to the cells. The re-generated *M*. *smegmatis* Δ*pep2*(u) Δ*glgE* and Δ*treS*(u) Δ*glgE* double mutants initially accumulated substantial amounts of M1P as revealed by TLC analysis (Fig 1C), before rapid spontaneous suppressor mutations occurred during sub-culturing likely due to insertional mutagenesis within the *glgC* locus as revealed by diagnostic PCR. Nevertheless, despite M1P accumulation, the *M*. *smegmatis* Δ*pep2*(u) Δ*glgE* double mutant was still resistant to trehalose (Fig 1D), in contrast to the *M*. *smegmatis* Δ*glgE* single mutant which is sensitive due to M1P hyperaccumulation in presence of trehalose [24]. This demonstrated that conversion of trehalose to M1P was efficiently blocked in this strain and that M1P synthesis must have occurred through a route independent of trehalose as a precursor.

### GlgC and GlgA are involved in the alternative route to M1P in *M*. *smegmatis*

As described above, deletion of the *glgE* gene causes genetic instability due to the toxicity of M1P, complicating the analysis of genes involved in alternative routes to M1P. We therefore established conditional *glgE* silencing in *M*. *smegmatis* in various genetic backgrounds in order to allow M1P accumulation only under controlled conditions. For this, the *glgE* gene was brought under control of a repressible promoter enabling gene silencing in the presence of anhydrotetracycline (ATc) (i.e. a tet-off system). Silencing of *glgE* in the conditional *M*. *smegmatis* c-*glgE*-tet-off mutant in presence of ATc resulted in M1P accumulation thereby reproducing the initial phenotype of the *M*. *smegmatis* Δ*glgE* gene deletion mutant (Fig 1E). Next, we analyzed the effect of conditional *glgE* gene silencing in various *M*. *smegmatis* mutant backgrounds. As expected from our analysis above, *glgE* silencing in the *M*. *smegmatis* Δ*treS*(u) c-*glgE*-tet-off mutant resulted in M1P accumulation, as revealed by TLC (Fig 1E). By contrast, synthesis of this phosphosugar was completely blocked in the *M*. *smegmatis* Δ*treS*(u) Δ*glgC*(u) c-*glgE*-tet-off strain. This corroborated our findings with the *glgC* suppressor mutation.

The phenotypes of the mutant strains implied that either GlgC or a downstream enzyme could produce M1P. It has already been shown that GlgC from *M*. *tuberculosis* efficiently converts glucose 1-phosphate (G1P) and ATP to ADP-glucose and pyrophosphate [25] with no indication of M1P being formed, according to ^1^H NMR spectroscopy. Another enzyme was therefore likely to be responsible for the formation of M1P. Since GlgC produces the ADP-glucose donor substrate for glycogen synthase GlgA, we then tested the involvement of GlgA in M1P synthesis. As for *glgC*, inactivation of the *glgA* gene fully abolished M1P production in the *M*. *smegmatis* Δ*treS*(u) Δ*glgA*(u) c-*glgE*-tet-off strain upon *glgE* silencing (Fig 1E). These observations demonstrate that the alternative route to M1P synthesis in *M*. *smegmatis* requires both ADP-glucose production by GlgC as well as GlgA enzyme activity.

### GlgP is not involved in the alternative route to M1P synthesis

Since GlgA is believed to be a glycogen synthase, we initially hypothesized that M1P production might arise through the phosphorolysis of α-glucans produced by the GlgC-GlgA pathway. GlgP is annotated as a glycogen phosphorylase that generates G1P. Since the gene of the glycogen phosphorylase GlgP is clustered with *glgE*, we speculated that GlgP could perhaps generate M1P instead. However, recombinant *M*. *tuberculosis* GlgP was only capable of forming G1P from glycogen and inorganic phosphate with no indication of M1P being formed, according to ^1^H NMR spectroscopy (S2A Fig). Furthermore, silencing of *glgE* in the absence of GlgP in the conditional *M*. *smegmatis* Δ*treS*(u) Δ*glgP*(u) c-*glgE*-tet-off mutant still resulted in M1P accumulation (S2B Fig). These observations prove that GlgP is a G1P-producing glycogen phosphorylase with no involvement in the alternative route to M1P production.

### Mycobacterial GlgA is an M1P synthesizing glucosyltransferase with low glycogen synthase activity

GlgA homologues from microorganisms are assumed to be glycogen synthases capable of elongating the non-reducing ends of glycogen using ADP-glucose as a donor (EC 2.4.1.21 ADP-α-D-glucose:(1→4)-α-D-glucan 4-α-D-glucosyltransferase) [26]. Indeed, such activity has been reported for *M*. *tuberculosis* GlgA [25]. We confirmed that the enzyme is capable of catalyzing this reaction with rabbit liver glycogen using a spectrophotometric assay that monitors the formation of ADP (Table 1). The *k*_cat_ (0.090 ± 0.007 s^-1^) was consistent with values reported previously (0.15 ± 0.01 s^-1^) [25]. However, such values are extremely low compared with those associated with *bona fide* glycogen synthases (e.g. human glycogen synthase 18.7 ± 0.3 s^-1^ [27]; *E*. *coli* glycogen synthase 694 ± 28 s^-1^ [28]). We therefore repeated the experiment with some bacterial α-glucan isolated from *Streptomyces venezuelae* [13, 29], which also belongs to the order *Actinomycetales*. However, the value of *k*_cat_ was even lower at 0.014 ± 0.008 s^-1^. Consistent with this, the specific activity of the *M*. *smegmatis* enzyme has been reported to be of the same order with glycogen isolated from *M*. *smegmatis* [30]. These rates would appear to be too low to be physiologically relevant [31], implying GlgA can play only a minor direct role for α-glucan formation in *M*. *tuberculosis*.

**Table 1:** GlgA activity with glycogen and α-glucan. Michaelis-Menten constants ± SE for recombinant *M*. *tuberculosis* GlgA with rabbit liver glycogen and *S*. *venezuelae* α-glucan were determined in triplicate in the presence of 1 mM ADP-glucose by monitoring the production of ADP with a spectrophotometric assay.

Acceptor	*k*_cat_ (s^-1^)	*K*_m_ (mg ml^-1^)	*V*_max_ (U mg^-1^)
Rabbit liver glycogen	0.090 ± 0.007	0.9 ± 0.2	0.12 ± 0.01
*S*. *venezuelae* α-glucan	0.014 ± 0.008	0.4 ± 0.1	0.02 ± 0.01

**Fig 2:**
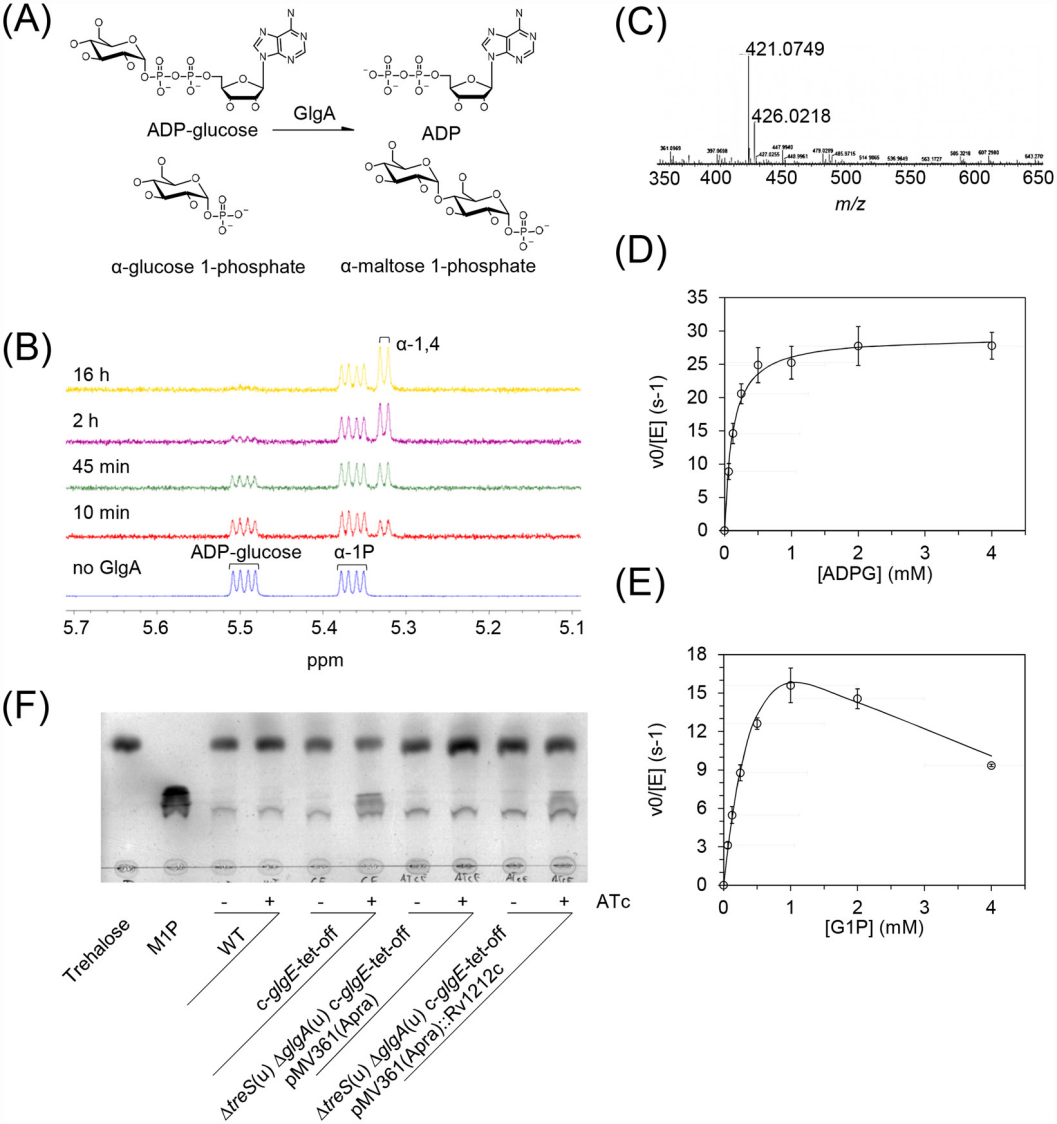
Mycobacterial GlgA is an M1P synthesizing glucosyltransferase. (A) The preferred reaction catalyzed by GlgA from *M*. *tuberculosis*. (B) Recombinant *M*. *tuberculosis* GlgA converts ADP-glucose and G1P to M1P. ^1^H NMR spectroscopy was used to monitor the anomeric protons of ADP-glucose and G1P (1 mM each) together with signals associated with the product in the presence of GlgA. The spectra show the concomitant consumption of ADP-glucose (~5.5 ppm) and appearance of resonances consistent with the formation of α-1,4 glycosidic linkages (~5.32 ppm). Given the lack of any resonances associated with free reducing ends (~5.1 ppm) and the retention of those associated with an α-glucosyl phosphate residue (~5.36 ppm), these observations are consistent with the formation of M1P. (C) Mass spectrometry of the product of the *M*. *tuberculosis* GlgA reaction with ADP-glucose and G1P. The dominant ion in the spectrum is consistent with the presence of M1P (*m/z* 421.0749 observed with 421.0752 expected for [M-H]^-^). The next most abundant ion is consistent with the presence of the co-product, ADP (*m/z* 426.0218 observed with 426.0221 expected for [M-H]^-^). (D) The dependence of *M*. *tuberculosis* GlgA activity on ADP-glucose. GlgA activity was determined spectrophotometrically by monitoring the production of ADP. A representative triplicate dataset with 1 mM G1P is shown as means and SEM. The data conform to the Michaelis-Menten eq 1 (fit shown as the solid line giving *r* ^2^ = 0.82). (E) The dependence of *M*. *tuberculosis* GlgA activity on G1P. GlgA activity was determined spectrophotometrically by monitoring the production of ADP. A representative triplicate dataset with 0.1 mM ADP-glucose is shown as means and SEM. Significant inhibition by G1P at concentrations >1 mM is apparent and the dataset conforms to a simple substrate inhibition eq 2 (fit shown as the solid line giving *r* ^2^ = 0.99). (F) *M*. *tuberculosis* GlgA (Rv1212c) was heterologously expressed in the *M*. *smegmatis* Δ*treS*(u) Δ*glgA*(u) c-*glgE*-tet-off mutant. Cells were cultivated for 24 h with or without 1 μg ml^-1^ ATc as indicated, and hot water extracts from 1 ml culture aliquots (normalized to OD_600 nm_ = 0.5) were analyzed by TLC. Conditional silencing of the *glgE* gene results in M1P accumulation, demonstrating that *M*. *tuberculosis* GlgA synthesizes M1P *in vivo*.

In light of the low glycogen synthase activity and its involvement in the alternative route to M1P, we then tested whether GlgA might be capable of synthesizing M1P directly using ADP-glucose as a donor with G1P as an acceptor (Fig 2A). Using NMR spectroscopy, it was immediately apparent that GlgA consumed ADP-glucose (double doublet at ~5.5 ppm) with the concomitant formation of a product with an α-1,4 linkage (doublet at ~5.32 ppm). Integration of peaks at the end of the reaction suggested the equilibrium position was ~1:8 in favour of the formation of the α-1,4 linkage from the nucleotide diphospho sugar. This is consistent with the thermodynamics of equivalent chemical equilibria [23]. The resonance associated with the phosphorylated α-anomeric position of G1P remained unchanged (double doublet at ~5.36 ppm) and yet no new reducing ends associated with glucose, maltose or higher oligomers were liberated (~5.15 ppm). Given that the resonance of the anomeric position of G1P is essentially identical to the corresponding position of M1P [16], it can be deduced that the product of the reaction was M1P (Fig 2B). High resolution mass spectrometry confirmed the presence of an ion consistent with M1P and the absence of ions associated with maltotriose phosphate or any higher oligomer (Fig 2C).

The substrate specificity was then explored further. A number of potential acceptors (1 mM) were tested using NMR spectroscopy to monitor the loss of ADP-glucose. The relative activities were G1P > glycogen > maltodextrin > maltoheptaose. No activity was detected with either glucose (up to 10 mM), glucose 6-phosphate, maltotetraose or isopannose. The lack of activity with glucose and glucose 6-phosphate emphasizes the importance of the phosphoryl group at the anomeric position in the specificity of the enzyme towards its preferred acceptor substrate, G1P. Interestingly, the enzyme also used UDP-glucose as a donor with G1P. However, it was less efficient than with ADP-glucose because the *k*_cat_ was ~ten-fold lower and the *K*_m_ for UDP-glucose was ~25-fold higher (with 1 mM G1P) giving a *k*_cat_/*K*_m_ ~250-fold lower. Surprisingly, UDP-glucose was not used as a donor when glycogen was used as the acceptor, as reported earlier [25], further restricting the ability of the enzyme to generate glycogen.

The kinetics of the formation of M1P by GlgA with ADP-glucose and G1P were then studied using the spectrophotometric assay. Most importantly, the values of *k*_cat_ were up to three orders of magnitude higher than for the glycogens. In addition, the values of *K*_m_ were ≤1 mM, which is typical for carbohydrate substrates of enzymes. GlgA activity as a function of ADP-glucose conformed to the Michaelis-Menten eq 1 at all concentration of G1P tested (Fig 2D and Table 2). GlgA activity was inhibited at high G1P concentrations (Fig 2E), which conformed to a substrate inhibition model defined by eq 2. Control experiments showed that this phenomenon was not due to an artefact from inadequate pH buffering capacity or limiting Mg^2+^ ions because the values of *K*_m_ and *K*_i_ were independent of buffer ion and Mg^2+^ concentrations. Interestingly, *k*_cat_ was nevertheless stimulated by Mg^2+^. The free Mg^2+^ concentration in the cytosol of *Escherichia coli* has been determined to be between 1 and 2 mM [32], so the values of *k*_cat_ shown in Table 2 with 5 mM Mg^2+^ would reasonably reflect those *in vivo*. Within error, the *K*_m_ and *K*_i_ for glucose 1-phosphate were very similar (~1 mM) and independent of the ADP-glucose concentration. This is consistent with the catalytic and inhibitory binding sites for G1P being one and the same. With a rising fixed G1P concentration, the *K*_m_ of ADP-glucose decreased while the *V*_max_ increased before it decreased. The former is consistent with the binding of G1P promoting the affinity of the enzyme for ADP-glucose in a ternary complex. The latter again shows substrate inhibition occurring with G1P at high concentrations. All of these properties are defining features of a compulsory-order ternary-complex mechanism whereby ADP-glucose binds to the enzyme before glucose 1-phosphate [33]. In such cases, substrate inhibition would be expected to occur when glucose 1-phosphate binds to the enzyme before the ADP co-product is released in the final step of the catalytic cycle, leading to the formation of a non-productive complex. Substrate inhibition by glycogen is presumably not observed at the concentrations tested because it is a poor substrate that does not bind sufficiently before ADP dissociates from the enzyme in the last step of the normal catalytic cycle.

**Table 2:** Dependence of GlgA activity on ADP-glucose and G1P. Kinetic constants ± SE for GlgA with ADP-glucose and G1P were determined in triplicate using the spectrophotometric assay monitoring the production of ADP. Data where ADP-glucose and G1P concentrations were varied were fitted to the Michaelis-Menten equation either without or with substrate inhibition, respectively.

Fixed [Substrate] (mM)	*k*_cat_ (s^-1^)	*K*_m_ (mM)	*K*_i_ (mM)	*V*_max_ (U mg^-1^)
**glucose 1-phosphate**	**ADP-glucose**			
0.25	20.0 ± 0.6	0.145 ± 0.020		27.7 ± 0.8
1.0	29.2 ± 1.3	0.121 ± 0.025		40.5 ± 1.8
2.5	19.8 ± 0.4	0.063 ± 0.006		27.5 ± 0.6
4.0	15.4 ± 0.3	0.046 ± 0.004		21.4 ± 0.4
**ADP-glucose**	**glucose 1-phosphate**			
0.1	39.0 ± 6.8	0.816 ± 0.204	1.450 ± 0.400	54.2 ± 9.4
0.5	97.9 ± 24.6	1.290 ± 0.400	0.578 ± 0.185	136.0 ± 34.0
4.0	80.6 ± 3.7	0.695 ± 0.045	0.895 ± 0.060	113.0 ± 6.0

In order to prove that *M*. *tuberculosis* GlgA (Rv1212c) also produces M1P *in vivo*, we heterologously expressed its gene in the *M*. *smegmatis* Δ*treS*(u) Δ*glgA*(u) c-*glgE*-tet-off mutant under control of the constitutive HSP60 promoter. While no M1P is formed in this conditional mutant upon silencing of the *glgE* gene, heterologous expression of *M*. *tuberculosis glgA* restored M1P accumulation (Fig 2F). This demonstrates that GlgA from *M*. *smegmatis* and *M*. *tuberculosis* are functionally equivalent and that *M*. *tuberculosis* GlgA is capable of synthesizing M1P *in vivo* while likely producing little or no classical glycogen.

### Compensatory flux of ADP-glucose links the GlgC-GlgA and TreS-Pep2 routes to M1P and α-glucan production

**Fig 3:**
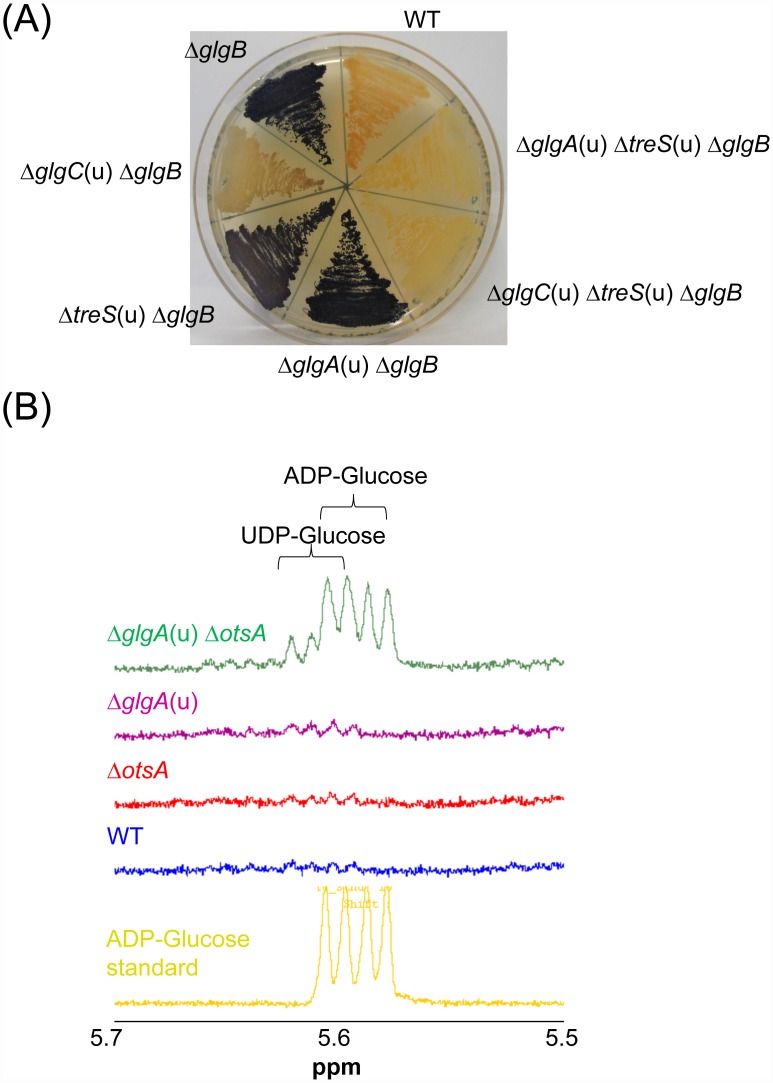
Compensatory flux of ADP-glucose through GlgA and OtsA links the GlgC-GlgA and TreS-Pep2 routes for α-glucan production. (A) α-Glucan visualization in *M*. *smegmatis* mutant strains. Cells were cultivated on Middlebrook 7H10 agar plates for 3 days and exposed to iodine vapor for staining of α-glucans. Branched α-glucans give a pale red-brown color. In the absence of branching enzyme GlgB, long linear glucans are produced resulting in a dark blue color of cells with the intensity of staining correlating with the amount of total cellular α-glucans. (B) Analysis of nucleotide sugar diphosphates in cell extracts of *M*. *smegmatis* mutant strains. Cells were cultivated for 24 h in Middlebrook 7H9 liquid medium. Due to trehalose auxotrophy of the *M*. *smegmatis* Δ*glgA*(u) Δ*otsA* mutant, 50 μM trehalose was added to all cultures. Cell suspensions were normalized to OD_600 nm_, washed with PBS, concentrated 50-fold and disrupted by bead beating. Cell-free extracts were heat inactivated for 15 min at 100°C, and ^1^H NMR spectroscopy was used to detect the anomeric protons of ADP-glucose.

Our observations on the function of GlgA imply that mycobacteria make little or no glycogen *via* the classical biosynthesis route, while suggesting a central role of GlgE for α-glucan production in mycobacteria. In order to assess to what extent the two alternative M1P pathways contribute to α-glucan biosynthesis, we analyzed different mutant strains of the model organism *M*. *smegmatis*. One advantage of this species is that deletion of the *glgB* gene is not lethal. This is because, unless trehalose is present in the growth medium, M1P does not build up to levels that are toxic, in contrast to *M*. *tuberculosis* [16]. The absence of the branching enzyme GlgB leads to the formation of very long insoluble linear α-glucans, which can be readily visualized using iodine vapor yielding dark blue inclusion complexes [34]. Mass spectrometry of cell free crude extracts showed small amounts of malto-oligosaccharides also accumulated in the mutant strain. These oligomers comprised 3–18 glucose rings, too short to stain blue with iodine. Nevertheless, the majority of the α-glucan was polymeric. We then deleted *glgB* in various *M*. *smegmatis* genetic backgrounds and stained mutant cells grown on agar plates (Fig 3A). Inactivation of GlgC led to a strong reduction in glucan content, while the lack of TreS had only a small effect, indicating that the GlgC-GlgA pathway is the dominant route of M1P synthesis in *M*. *smegmatis* under the conditions tested. Combined inactivation of both GlgC and TreS resulted in, what appeared to be, a complete absence of α-glucan. In stark contrast to the profound impact of losing GlgC, it was striking that the loss of GlgA had no discernible effect on α-glucan content. However, while mutation in either *treS* or *glgA* had only a small influence on glucan production, their simultaneous inactivation in a Δ*glgB* strain completely blocked α-glucan production. This is consistent with the existence of the two routes for M1P synthesis that diverge after GlgC.

*M*. *tuberculosis* GlgA is able to use both ADP-glucose and UDP-glucose as donors in the synthesis of M1P. The ability to use these two nucleotide sugar diphosphates is a recurring theme in trehalose and α-glucan metabolism in *M*. *tuberculosis*, because trehalose 6-phosphate synthase OtsA also uses both [25]. Since OtsA is involved in the biosynthesis of trehalose as a substrate for the TreS-Pep2 pathway [18], we speculated that a compensatory rechanneling of ADP-glucose and/or UDP-glucose between OtsA and GlgA occurs. We therefore analyzed extracts obtained from the *M*. *smegmatis* Δ*glgA* and Δ*otsA* single and Δ*glgA*(u) Δ*otsA* double mutants for the presence of nucleotide sugar diphosphates by ^1^H NMR spectroscopy. While the accumulation of ADP-glucose, and likely some UDP-glucose, was detected in the *M*. *smegmatis* Δ*glgA*(u) Δ*otsA* double mutant, none was detected in the single mutants (Fig 3B). These findings are consistent with the flux of ADP-glucose being redirected through OtsA when GlgA is inactive. This likely leads to an enhanced generation of trehalose and subsequent conversion to M1P *via* the TreS-Pep2 pathway, explaining why a lack of GlgA alone has little effect on glucan content. Accumulation of ADP-glucose further implies that, apart from GlgA and OtsA, no other enzymes significantly consume this nucleotide sugar diphosphate in *M*. *smegmatis*.

Semiquantitative dot blot analyses of cellular and capsular glucan preparations from mutant cells using a glucan-specific monoclonal antibody corroborated the dominant role of GlgC over TreS for glucan production in *M*. *smegmatis*. Moreover, this revealed that mutations had a similar impact on both cytosolic and capsular glucan content, with combined inactivation of GlgC and TreS necessary to completely block biosynthesis of both polymers (S3B Fig). The equivalent impact on both cytosolic and capsular α-glucans implies synthesis occurs intracellularly with some of the polymer being exported to the capsule.

### The GlgE pathway mediates both intracellular and capsular α-glucan production in *M*. *tuberculosis*

The observations described so far showed that α-glucan production in *M*. *smegmatis* relies on the GlgE pathway, and that the M1P substrate can be provided by two alternative routes, with the GlgC-GlgA branch being dominant in this organism under the tested growth conditions.

**Fig 4:**
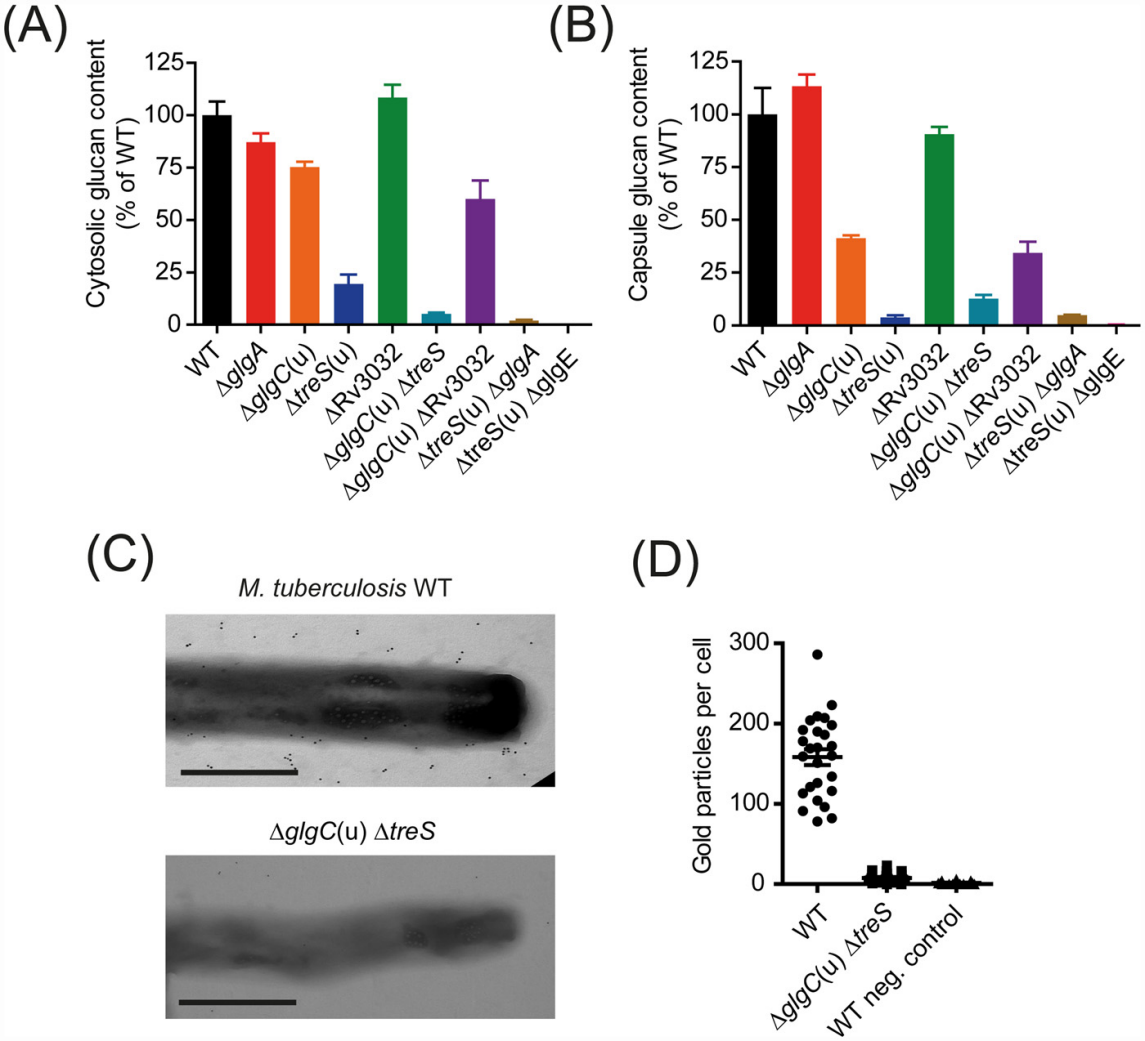
The central importance of the GlgE pathway in intracellular and capsular α-glucan synthesis in *M*. *tuberculosis*. Quantification of intracellular (A) or extracellular (i.e. capsular) (B) α-glucan in *M*. *tuberculosis* H37Rv mutant strains. Cells were grown in Middlebrook 7H9 liquid medium for 7 days with shaking. Intracellular glucans were measured in hot water extracts of washed cells. Capsular glucans were measured from cell-free culture supernatants. Intracellular and capsular glucans were assayed by sandwich ELISA employing an α-glucan specific monoclonal antibody. Similar results for intracellular glucan content were also obtained using an enzymatic assay with cells from independent biological replicates (S3 Fig). Values were normalized based on OD 600 nm of cultures. Values in (A) and (B) represent means of triplicates ± SEM. (C) Visualization of the α-glucan capsule in the *M*. *tuberculosis* WT and the Δ*glgC*(u) Δ*treS* mutant by immunogold labelling. Cells were grown in liquid medium without shaking, fixed, labelled with an α-glucan specific monoclonal antibody, and analyzed by electron microscopy (scale bar 0.5 μm). (D) Quantitative evaluation of α-glucan capsule visualization as shown in (C), plotted as anti-α-glucan specific gold particles per cell. Values represent means ± SEM (WT n = 27, Δ*glgC*(u) Δ*treS* n = 28). Negative controls were not treated with the primary anti-α-glucan antibody (n = 32).

In order to determine the individual contribution of the GlgC-GlgA and TreS-Pep2 branches of the GlgE pathway and of the Rv3032 pathway for α-glucan production in *M*. *tuberculosis*, we investigated a set of *M*. *tuberculosis* mutant strains and quantified intracellular and capsular α-glucans employing an enzymatic assay and/or by ELISA using a glucan-specific monoclonal antibody. In contrast to *M*. *smegmatis*, inactivation of GlgC resulted in only a moderate reduction of the intracellular and capsular glucan content, consistent with previous studies [10], while a lack of TreS had a profound impact (Fig 4A and 4B and S3B Fig). This indicates that under the tested culture conditions the TreS-Pep2 branch provides most of the M1P for the GlgE pathway in *M*. *tuberculosis* whereas the GlgC-GlgA branch is much less active. In fact, we could not detect M1P in the *M*. *tuberculosis* Δ*treS*(u) Δ*glgE* mutant using ^1^H NMR spectroscopy. Whole genome resequencing confirmed that this strain did not harbor any mutation in *glgC* or *glgA* that might block the alternative route to M1P, so any amount of M1P produced was below the limit of detection. As with *M*. *smegmatis*, inactivation of GlgA had a smaller influence on α-glucan levels compared to GlgC, suggesting that compensatory rechanneling of ADP-glucose between GlgA and OtsA might also occur in *M*. *tuberculosis*. However, in contrast to *M*. *smegmatis*, we were unable to generate the *M*. *tuberculosis* Δ*glgA*(u) Δ*otsA* double mutant despite repeated attempts, implying that combined inactivation is lethal, potentially due to accumulation of toxic levels of ADP-glucose. As with *M*. *smegmatis*, combined inactivation of either GlgC and TreS or GlgA and TreS resulted in mutants that were virtually devoid of α-glucans (Fig 4A and 4B and S3B Fig). The equivalent impact on both cytosolic and capsular α-glucans again implies synthesis occurs intracellularly with some of the polymer being exported to the capsule. Importantly, deletion of both *treS* and *glgE* also blocked the production of α-glucan, despite the presence of intact *glgC* and *glgA* genes. This emphasizes the fundamental importance of the GlgE pathway for the biosynthesis of both intracellular and capsular α-glucans in *M*. *tuberculosis* and is consistent with GlgA having no direct role in polymer synthesis (Fig 4A and 4B). To corroborate the requirement of the GlgE pathway for extracellular α-glucans, we analyzed cells by immunogold electron microscopy, proving that the *M*. *tuberculosis* Δ*glgC*(u) Δ*treS* mutant completely lacked capsular α-glucans (Fig 4C and 4D).

Rv3032 has previously been implicated in the formation of intracellular, but not capsular, α-glucan [10]. However, in contrast to the dominant role of the GlgE pathway, in our hands deletion of Rv3032 had no significant impact on the levels of either intracellular or capsular α-glucans regardless of the genetic backgrounds tested (Fig 4A and 4B and S3B Fig). In addition, the presence of Rv3032 did not rescue any of the α-glucan-deficient strains. Furthermore, the dispensability of Rv3032 for α-glucan formation in *M*. *tuberculosis* was supported by the finding that the *M*. *tuberculosis* Δ*glgC*(u) Δ*otsA* mutant displayed trehalose auxotrophy (S4 Fig). In the absence of OtsA, mycobacteria can produce trehalose only from internal α-glucans *via* the TreX-TreY-TreZ pathway [18], implying that the *M*. *tuberculosis* Δ*glgC*(u) Δ*otsA* mutant is essentially free of α-glucans, despite Rv3032 being present. Furthermore, while Sambou et al. [10] postulated that combined inactivation of Rv3032 and GlgA is lethal, an *M*. *tuberculosis* Δ*glgA*(u) ΔRv3032 double mutant was viable in our hands and exhibited only a slight reduction in α-glucan content (S3B Fig). These findings strongly suggest that Rv3032 has little or no role in the synthesis of α-glucan.

### α-Glucan biosynthesis *via* the GlgE pathway is important for virulence of *M*. *tuberculosis* in mice

**Fig 5:**
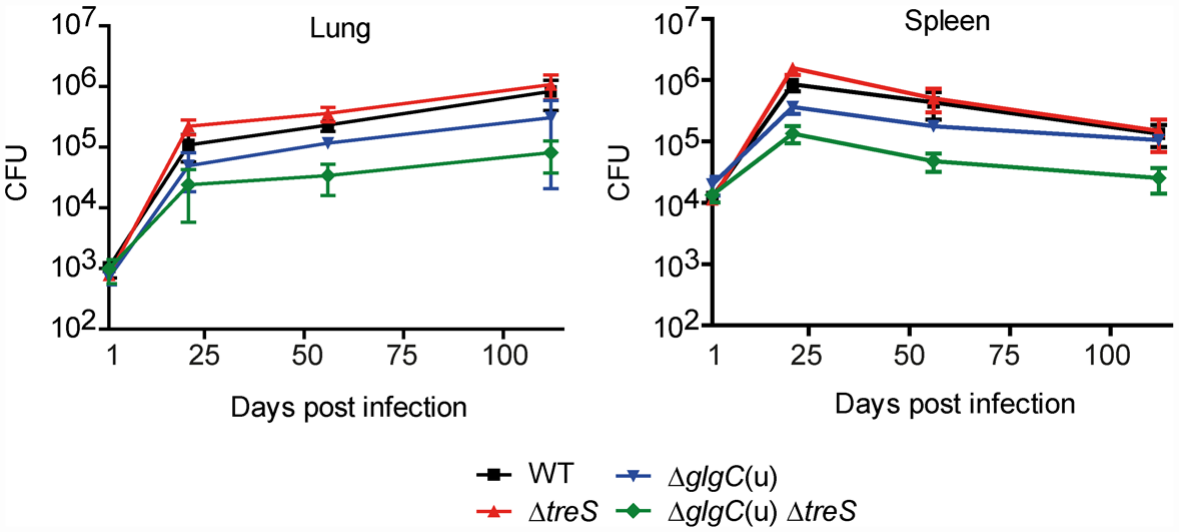
Importance of GlgE-mediated α-glucan synthesis for virulence of *M*. *tuberculosis* in mice. BALB/c mice were challenged by intravenous infections with 5 × 10^4^ colony forming units of *M*. *tuberculosis* α-glucan mutant strains (mean ± SD, n = 3 per time point). The growth of the double mutant was significantly attenuated in lung and spleen as compared to wild-type at all time-points (*p* < 0.05), except for the earliest 21 days post-infection time point in the lung.

Sambou and colleagues have reported that a *glgA* mutant was impaired in its ability to persist in mice and linked this to a somewhat lower level of capsular α-glucan [10]. However, the full extent to which α-glucan is important for the virulence of *M*. *tuberculosis* remained unclear because they were unable to generate an α-glucan-deficient strain. Having now elucidated the configuration of pathways underlying α-glucan biosynthesis, it was possible, for the first time, to test the full impact of α-glucan on *M*. *tuberculosis* pathogenesis in mice employing rationally designed α-glucan-deficient mutants. The *M*. *tuberculosis* Δ*glgC*(u) Δ*treS* mutant was significantly attenuated for growth in the lung and spleen of BALB/c mice during both the acute and chronic phase of infection (Fig 5). This is in large agreement with the phenotype reported for the *glgA* mutant but differs in that this single mutant was specifically attenuated only during the chronic infection phase [10]. Genome sequencing confirmed the genotype of the double mutant strain and a lack of secondary mutations, providing unequivocal evidence that α-glucan is indeed required for the full virulence of the wild-type strain. Interestingly, deletion of *treS* alone had no effect in a mouse model (Fig 5). This implies that while the TreS/Pep2-dependent route for M1P synthesis dominates in *M*. *tuberculosis* in culture, the pathways are subject to regulation [25, 35] such that the GlgA-dependent route is more important during infection consistent with previous work [10]. However, only combined inactivation of both M1P-synthesis routes can effectively block glucan formation throughout infection.

## Discussion

**Fig 6:**
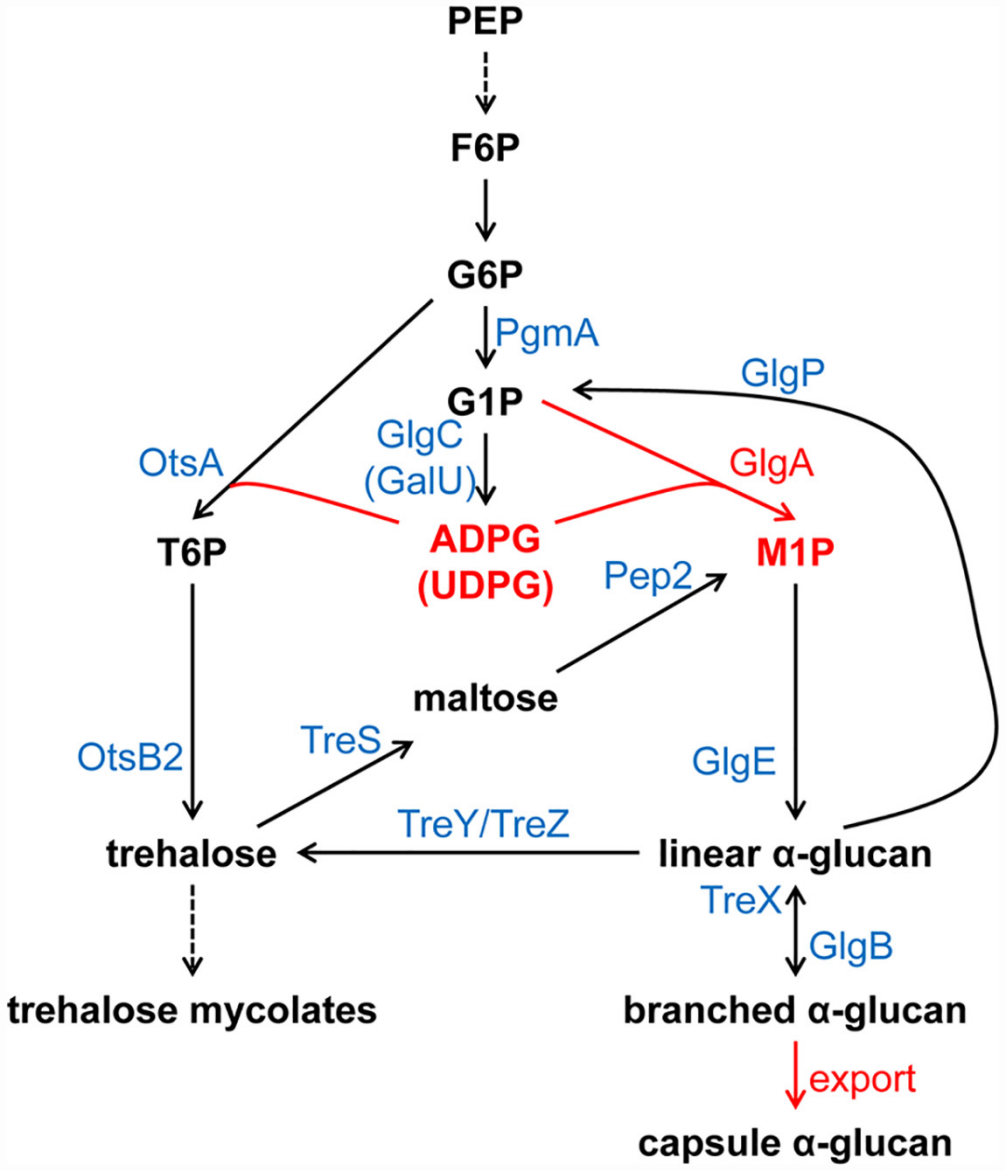
Revised model of GlgE-mediated intracellular and capsular α-glucan synthesis in mycobacteria. The M1P building block for the maltosyltransferase GlgE is formed *via* two alternative routes, TreS-Pep2 and GlgC-GlgA. Both routes are connected *via* the shared use of ADP-glucose by GlgA and OtsA, the latter providing the trehalose substrate for the TreS-Pep2 pathway. GlgA, like OtsA, is also capable of using UDP-glucose as a donor, which in turn is produced from G1P by GalU, but this appears to be less significant *in vivo*. GlgE is essential for intracellular and capsular α-glucan synthesis and generates linear maltooligosaccharides as the substrates for the branching enzyme GlgB. α-Glucans are produced intracellularly with partial coupling to secretion by unknown transport mechanisms. Steps highlighted in red are new findings as described in this report. G6P, glucose 6-phosphate; G1P, glucose 1-phosphate; M1P, α-maltose 1-phosphate; T6P, trehalose 6-phosphate; ADPG, ADP-glucose; UDPG, UDP-glucose.

Prior to this study, it was thought that three partially redundant routes collectively contribute to α-glucan production in *M*. *tuberculosis* and other mycobacteria: the GlgC-GlgA pathway for classical glycogen, the recently discovered TreS-Pep2-GlgE pathway for trehalose-to-glucan conversion, and the Rv3032 pathway. This multiplicity of pathways for the production of one type of molecule was puzzling, so it was unclear how these pathways interrelate in the biosynthesis of intracellular and capsular α-glucans. Refuting previous assumptions, we have now demonstrated that both cytosolic and capsular α-glucan polymers in *M*. *tuberculosis*, *M*. *smegmatis* and probably all other mycobacteria are predominantly, if not exclusively, synthesized by the maltosyltransferase GlgE together with the branching enzyme GlgB. In addition, the activated M1P donor substrate of GlgE is unexpectedly generated by two alternative routes: TreS-Pep2 as described previously [16] and GlgC-GlgA (Fig 6). These findings on the central role of the GlgE pathway in global glucan production in mycobacteria support our very recent observation that purified recombinant GlgE and GlgB together, using M1P as the sole substrate, are sufficient to generate α-glucans *in vitro* possessing the same distinctive structural features as the polymers isolated from the cytosol and capsule of *M*. *tuberculosis* [13].

While mycobacterial GlgA was believed to be a glycogen synthase, we have shown that GlgA from *M*. *tuberculosis* is a M1P-producing glucosyltransferase three orders of magnitude more efficient at transferring glucose from ADP-glucose to glucose 1-phosphate than to glycogen. By comparing the rates of reaction with a series of potential acceptors, it is apparent that maltooligosaccharides are even poorer substrates than glycogen, highlighting that the +2 sub-site of GlgA has a clear preference for an α-1-phosphate group. It would therefore be more appropriate to define the enzyme as an ADP-α-D-glucose:α-D-glucose-1-phosphate 4-α-D-glucosyltransferase (EC 2.4.1). Furthermore, since mycobacterial GlgA likely produces little or no classical glycogen *in vivo*, the name GlgA appears no longer to be biologically relevant and we propose to call the enzymes with this new activity GlgM in future publications.

GlgA from *M*. *tuberculosis* is a GT4 family enzyme according to the CAZy database [36] (http://www.cazy.org/) and is predicted to have a GT-B fold and be a retaining enzyme. We have now confirmed that it is an α-retaining enzyme. Such glycosyltransferases are thought to use an S_N_ *i*-type catalytic mechanism [37]. Indeed, it has recently been reported that trehalose 6-phosphate synthase OtsA, a UDP-glucose:D-glucose-6-phosphate 1-α-D-glucosyltransferase (EC 2.4.1.15) and member of the GT20 family, uses an S_N_ *i* mechanism and kinetic and structural evidence supports the compulsory binding of UDP-glucose before glucose 6-phosphate [38, 39]. It seems likely that GlgA from *M*. *tuberculosis* shares these properties because it is predicted to also have a GT-B fold, catalyzes the same underlying chemistry and uses a compulsory-order ternary-complex mechanism. Interestingly, substrate inhibition of GlgA could help limit accumulation of M1P when G1P levels were high. Importantly, experimentally characterized and, to the best of our knowledge, *bone fide* glycogen synthases [26] also share a GT-B fold and are classified as either GT3 or GT5 family enzymes depending on whether they come from eukaryotes or prokaryotes, respectively [36]. All GT5 family enzymes characterized to date are glycogen synthases. This contrasts with the GT4 family membership of *M*. *tuberculosis* GlgA. We extended our recent bioinformatic analysis [40] and identified that GT4 and GT5 family membership of apparent glycogen synthase homologues can be a feature of bacteria whether they are either Gram positive or negative and whether the *glgA* gene coexists with *glgE* in a given genome or not in all combinations with one exception. It is striking that in every case where these two genes coexist in Gram positive bacteria, the GlgA belongs exclusively to the GT4 family and never to the GT5 family. The coexistence of the genes is consistent with the new activity being associated with the alternative route for the generation of M1P for the GlgE pathway in this clade. These bacteria are typically actinomycetes including mycobacteria and streptomycetes. Overall, ~32% of the GlgA homologues are GT4 members, implying that the proportion of microbes that possess the classical glycogen pathway is ~20%, a value lower than previously estimated [40].

The two alternative pathways for generating the M1P building block for GlgE-mediated α-glucan production in mycobacteria, TreS-Pep2 and GlgC-GlgA, are linked *via* the shared use of the intermediate ADP-glucose by GlgA and OtsA (Fig 6). GlgA and OtsA can also use UDP-glucose as donors *in vitro* in the synthesis of M1P or trehalose 6-phosphate, respectively [25]. However, the phenotypes of mutant strains suggest that ADP-glucose is the main donor *in vivo* under the used growth conditions because little, if any, UDP-glucose accumulated in the *M*. *smegmatis* Δ*glgA*(u) Δ*otsA* double mutant. UDP-glucose might instead be primarily used for production of arabinogalactan, *via* the essential UDP-galactose 4´-epimerase GalE1 (MSMEG_6142, homologue of Rv3634c in *M*. *tuberculosis* H37Rv), which converts UDP-glucose to UDP-galactose. In any case, such donor promiscuity might provide metabolic plasticity and the opportunity for additional levels of regulation. The flux of ADP-glucose seems to be sufficiently redirected such that the net rate of M1P generation and α-glucan accumulation can be balanced to some extent when one of the two routes is perturbed. However, the two routes are obviously subject to regulation such that the TreS-Pep2 pathway dominates in *M*. *tuberculosis* in culture while the GlgA-dependent route is more important during infection. The molecular basis underlying these regulatory mechanisms under different physiological conditions remains to be fully elucidated.

Our data show that the Rv3032 pathway does not contribute substantially, if at all, to α-glucan production in mycobacteria under the tested culture conditions. Rather, its function appears to be restricted to the synthesis of specialized oligomeric α-glucan derivatives such as MGLPs [15]. This contrasts with another report claiming that an Rv3032 gene deletion mutant of *M*. *tuberculosis* exhibited a somewhat reduced intracellular, but not capsular, glucan content [10]. This might be due to use of different culture conditions or strains used. Although our data cannot rule out that Rv3032 uses ADP-glucose and/or UDP-glucose to some extent for the synthesis of glycogen-like α-glucans under certain circumstances, this seems unlikely in light of our findings. Nevertheless, given the new specificity of *M*. *tuberculosis* GlgA, we can almost certainly rule out a previously proposed functional redundancy between *M*. *tuberculosis* GlgA and Rv3032. In fact, we could successfully generate an *M*. *tuberculosis* Δ*glgA*(u) ΔRv3032 double mutant, clearly refuting the hypothesized synthetic lethality between GlgA and Rv3032 [10]. In addition, the *M*. *tuberculosis* Δ*glgA*(u) ΔRv3032 double mutant still produced α-glucan in culture.

Our study demonstrates that the GlgE pathway produces both intracellular and capsular α-glucans in *M*. *tuberculosis*, although separate biosynthetic routes had previously been postulated [10]. This implies that capsular α-glucans have an intracellular biosynthetic origin that necessitates the presence of dedicated transporters for α-glucan secretion, which have yet to be identified. Indeed, our model is in accord with the observation that the cytosolic and capsular materials from *Mycobacterium bovis* [12] and *M*. *tuberculosis* [13] have very similar physicochemical properties, consistent with a common biosynthetic origin.

A previous report that an *M*. *tuberculosis glgA* mutant was impaired in virulence [10] must now be reinterpreted as a block in the production of the donor substrate of GlgE rather than in the formation of the polymer. Furthermore, our new understanding of the metabolic network has established why blocking GlgA alone cannot completely prevent polymer synthesis, because the TreS-Pep2 route can compensate in the supply of M1P for GlgE. It was therefore necessary to generate double mutants to create the first strains devoid of α-glucan. This was achievable with several combinations of genes in a rational way. Importantly, we have demonstrated that such an *M*. *tuberculosis* strain devoid of α-glucan is less virulent than the wild-type providing the first evidence directly linking the GlgE pathway with virulence.

This work provides the first complete understanding of the metabolic network associated with formation of α-glucan in mycobacteria. This has implications for the development of new therapies. GlgE is a genetically validated target [16, 41] that has already attracted attention [42]. Blocking GlgE leads to the toxic accumulation of M1P [16], so potential suppressor mutations in four genes (*treS*, *pep2*, *glgC*, *glgA*) associated with M1P formation could potentially rescue growth and limit the *in vivo* efficacy of GlgE inhibitors. However, the continued absence of α-glucan production in such suppressor mutants would still compromise the virulence of the pathogen, making GlgE an even more attractive target. The targeting of other enzymes in the pathways to block the production of α-glucan can now be done in a more rational manner. Similarly, the feeding of substrate analogues in labelling studies can be done in a more predictable way. Mutant strains blocked in individual pathways and/or devoid of α-glucan will also be a useful resource for future studies. Furthermore, attenuated *M*. *tuberculosis* strains that lack α-glucan could be better suited as a vaccine because not only might they be less able to evade immunity but other antigenic cell surface components might also be physically more exposed.

## Materials and Methods

### Strains and growth conditions

All strains were derived from *M*. *smegmatis* mc^2^155 and *M*. *tuberculosis* H37Rv and are listed in S1 and S2 Tables. Cells were grown aerobically at 37°C in Middlebrook 7H9 media supplemented with 0.5% (v/v) glycerol and 0.05% (v/v) Tyloxapol and containing 10% (v/v) ADS enrichment (5%, w/v, bovine serum albumin fraction V (BSA); 2%, w/v, glucose; 0.85%, w/v, sodium chloride). Hygromycin (50 mg l^-1^), kanamycin (20 mg l^-1^) and apramycin (10 mg l^-1^) were added for selection of appropriate strains.

### Generation of site-specific gene deletion mutants

Site-specific gene deletion mutants of *M*. *smegmatis* mc^2^155 and *M*. *tuberculosis* H37Rv were generated by specialized transduction employing temperature-sensitive mycobacteriophages essentially as described previously [43]. Briefly, for the generation of allelic exchange constructs for gene replacement with a γδ*res*-*sacB*-*hyg*-γδ*res* cassette comprising a *sacB* as well as a hygromycin resistance gene flanked by *res*-sites of the γδ-resolvase, upstream- and downstream-flanking DNA regions were amplified by PCR employing the oligonucleotides listed in S3 Table. Subsequently, the upstream and downstream flanks were digested with the indicated restriction enzymes, and ligated with *Van*91I-digested p0004S vector arms (= pYUB1471 [44]). The resulting knock-out plasmids were then linearized with *Pac*I and cloned and packaged into the temperature-sensitive phage phAE159 [44], yielding knock-out phages which were propagated in *M*. *smegmatis* at 30°C. Allelic exchange in *M*. *smegmatis* and *M*. *tuberculosis* using the knock-out phages was achieved by specialized transduction using hygromycin (50 mg l^-1^) for selection, resulting in gene deletion and replacement by the γδ*res*-*sacB*-*hyg*-γδ*res* cassette. For the generation of unmarked mutants, the γδ*res*-*sacB*-*hyg*-γδ*res* cassette was removed employing specialized transduction using the phage phAE280 expressing the γδ-resolvase [44] using sucrose (3%, w/v) for counterselection. All mutants were verified by Southern analysis of digested genomic DNA using appropriate restriction enzymes and probes. For the unmarked Rv1212c locus, an additional diagnostic PCR was performed employing primers binding outside the flanks used for allelic exchange, and the PCR product was sequenced confirming the correct genotype of the *M*. *tuberculosis* Δ*glgA*(u) and Δ*glgA*(u) ΔRv3032 mutants. Furthermore, whole genome sequencing was carried out for the *M*. *tuberculosis* Δ*glgC*(u) Δ*treS* and Δ*treS*(u) Δ*glgE* mutants to confirm their genotypes.

### Generation of the conditional *M*. *smegmatis* c-*glgE-*tet-off mutant

For establishing regulated expression of the *glgE* gene, a synthetic gene cassette (*hyg*-P*myc1*-4X*tetO*) comprising a hygromycin resistance gene and the P*myc1* promoter from *M*. *smegmatis* engineered to contain four *tetO* operator sites, which are the DNA binding sites for the cognate repressor protein TetR, was inserted immediately upstream of the *glgE* start codon in *M*. *smegmatis*. Targeted gene knock-in was achieved by specialized transduction employing temperature-sensitive mycobacteriophages essentially as described above for gene deletion mutants. Briefly, for the generation of allelic exchange constructs for site-specific insertion in *M*. *smegmatis* of the *hyg*-P*myc1*-4X*tetO* cassette, upstream- and downstream DNA regions flanking the *glgE* start codon were amplified by PCR employing the oligonucleotides listed in S3 Table. Subsequently, the upstream and downstream flanks were digested with the indicated restriction enzymes, and ligated with *Van*91I-digested pcRv1327c-4XtetO vector arms (sequence provided in S1 Text). The resulting knock-in plasmid was then linearized with *Pac*I and cloned and packaged into the temperature-sensitive phage phAE159 [44], yielding a knock-in phage which was propagated in *M*. *smegmatis* at 30°C. Allelic exchange in *M*. *smegmatis* using the knock-in phage at the nonpermissive temperature of 37°C was achieved by specialized transduction using hygromycin (50 mg l^-1^) for selection, resulting in site-specific insertion of the *hyg*-P*myc1*-4X*tetO* cassette. The *M*. *smegmatis* c-*glgE*-4X*tetO* knock-in mutant was verified by Southern analysis of digested genomic DNA using an appropriate restriction enzyme and probe. For achieving controlled gene expression of the target gene *glgE*, a synthetic gene (*revtetR*) derived from Tn10 *tetR* encoding a mutated TetR protein with reversed binding affinity to *tetO* sites upon binding of tetracycline [45] was heterologously expressed in the knock-in mutant. For this, the *revtetR* gene was amplified by PCR employing the oligonucleotide primer pair 5´-TTTTTGAATTCATGAGCACGATCCGCGGTACCATC-3´ and 5´-TTTTTAAGCTTAGGAGCCGCTCTCGCACTTCAG-3´ using the plasmid pTC-28S15-0X (Addgene plasmid 20316) as a template and cloned using the restriction enzymes *EcoR*I and *Hin*dIII (underlined) into the episomal *E*. *coli*-mycobacterium shuttle plasmid pMV261-RBS-G, which is a derivative of plasmid pMV261 [46] harboring a mutated ribosome binding site (sequence provided in S2 Text). The resulting plasmid pMV261::*revtetR*_RBS-G providing constitutive gene expression from the HSP60 promoter in mycobacteria was transformed by electroporation into the *M*. *smegmatis* c-*glgE*-4X*tetO* knock-in mutant using solid medium containing 50 mg l^-1^ hygromycin and 20 mg l^-1^ kanamycin for selection. This yielded the conditional mutant *M*. *smegmatis* c-*glgE*-4X*tetO* pMV261::*revtetR*_RBS-G (referred to as *M*. *smegmatis* c-*glgE*-tet-off mutant) allowing silencing of the *glgE* gene in presence of anhydrotetracycline (ATc).

### Genetic complementation of *M*. *smegmatis* mutants

The *M*. *tuberculosis glgC* gene was amplified by PCR employing the oligonucleotide primer pair 5´-TTTTTCAGCTGCAATGAGAGAAGTGCCGCACGTGCTG-3´ and 5´-TTTTTAAGCTTCTAGATCCAAACACCCTTGCCCAC-3´ using genomic DNA as a template and cloned using the restriction enzymes *Pvu*I and *Hind*III (underlined) into the integrative *E*. *coli*-mycobacterium shuttle plasmid pMV361(Kan) [46]. The resulting plasmid pMV361::Rv1213, providing constitutive gene expression from the HSP60 promoter in mycobacteria, was introduced by electroporation into the *M*. *smegmatis* Δ*glgE*(u) Δ*pep2 glgC*:IS*1096* mutant using solid medium containing 50 mg l^-1^ hygromycin and 20 mg l^-1^ kanamycin for selection. The *M*. *tuberculosis glgA* gene was amplified by PCR employing the oligonucleotide primer pair 5´-TTTTTTTAATTAAATGCGGGTGGCGATGTTGACTCG-3´ and 5´-TTTTTAAGCTTTTACGCGCACACCTTCCGGTAGATG-3´ using genomic DNA as a template and cloned using the restriction enzymes *Pac*I and *Hind*III (underlined) into the integrative *E*. *coli*-mycobacterium shuttle plasmid pMV361(Apra)-PacI, which is a derivative of plasmid pMV361 [46] harboring a *PacI* cloning site. The resulting plasmid pMV361(Apra)::Rv1212c providing constitutive gene expression from the HSP60 promoter in mycobacteria as well as the empty vector control were introduced by electroporation into the conditional *M*. *smegmatis* Δ*treS*(u) Δ*glgA*(u) c-*glgE*-tet-off mutant using solid medium containing 50 mg l^-1^ hygromycin, 20 mg l^-1^ kanamycin, and 10 mg l^-1^ apramycin for selection.

### Genome sequencing

Genomes were sequenced using an Illumina HiSeq 2500 next-generation sequencer (San Diego, CA, USA) and compared with the parent *M*. *tuberculosis* H37RvMA genome (GenBank accession GCA_000751615.1) [47]. Genomic DNA was extracted from bacterial cultures and prepared for sequencing using the standard paired-end genomic DNA sample prep kit from Illumina. Paired-end sequence data was collected with a read length of 106 bp. Base-calling was performed using Casava software, v1.8. The reads were assembled using a comparative genome assembly method. The mean depth of coverage was 188× (Δ*glgC*(u) Δ*treS*) and 246× (Δ*treS*(u) Δ*glgE*).

The deletion of the appropriate genes in the Δ*glgC*(u) Δ*treS* and Δ*treS*(u) Δ*glgE* mutants was confirmed. The only additional difference in the Δ*glgC*(u) Δ*treS* mutant was a single polymorphism giving rise to a synonymous silent mutation (L190L) in Rv2217 (*lipB*). The decreased virulence of this strain (Fig 5) is therefore clearly attributable to the loss the *glgC* and *treS* genes. The Δ*treS*(u) Δ*glgE* mutant had four additional mutations that appear unrelated to α-glucan metabolism. Rv1219c is a transcription factor whose regulon is currently unknown. There was a loss of CC 28 bp upstream (5') of this gene. Rv1739c is a sulfate ABC transporter in which there was a V524I mutation. Rv2402 is annotated as a hypothetical protein and there was a H621D mutation. PpsE is a component of the type I (modular) polyketide synthase involved in producing phenolphthiocerol dimycolate, a cell-wall glycolipid. There was a mutation giving rise to a loss of amino acid A567. It is not uncommon for random secondary mutations like these to occur in a genetically manipulated strain.

### Carbohydrate TLC analysis

Carbohydrates were extracted from equal amounts of cells with hot water (95°C for 4 h) and analyzed by TLC on Silica gel 60 (EMD Chemicals) using the solvent system 1-propanol:ethyl acetate:water (6:1:3, v/v/v). Substances were visualized by immersing TLC plates in 10% (v/v) sulfuric acid in ethanol followed by charring at 180°C for 10 min.

### Recombinant *M*. *tuberculosis* proteins

The *M*. *tuberculosis glgA* and *glgP* genes were synthesized with optimized codon usage for expression in *Escherichia coli* (Genscript Corporation, Piscataway, NJ), allowing their production with an *N*-terminal His_6_ tag and TEV cleavage site. The constructs were ligated into a pET21a expression vector (Novagen, Darmstadt, Germany) using *Nde*I and *Bam*HI restriction sites. For the production of GlgA, transformed *E*. *coli* SoluBL21 cells (AMS Biotechnology Europe Ltd) were grown at 18°C to an OD_600 nm_ of 0.6 in Lysogeny Broth and expression was induced with 0.5 mM isopropyl β-D-thiogalactopyranoside. Bacteria were harvested and lysed after a further 16 h of incubation. The enzyme was purified using a 1 ml HisTrap FF column (GE Healthcare, Amersham, United Kingdom) with an imidazole gradient followed by a Superdex S200 16/60 size exclusion chromatography column (Pharmacia Biotech, Amersham, United Kingdom) with 50 mM MOPS buffer, pH 8.0, containing 0.1 mM ethylenediaminetetraacetic acid, 5 mM MgCl_2_, 5% (w/v) sucrose, 10% (v/v) glycerol and 50 mM NaCl. GlgA-containing fractions were pooled and concentrated to ~1.5 mg l^-1^ and aliquots were stored at -80°C.

A similar strategy was used for the production of recombinant *M*. *tuberculosis* GlgP protein with modifications, which was produced in *E*. *coli* BL21 Star(DE3) cells (Novogen) grown at 18°C for 16 h in auto-induction medium [48] supplemented with 1% (v/v) ethanol. GlgP-containing fractions from Ni-affinity chromatography were dialyzed into 50 mM HEPES buffer, pH 7.5, and did not require further purification.

### Enzyme assays

GlgA activity was measured using a continuous enzyme-coupled spectrophotometric assay that monitors ADP release. Chemicals were purchased from Sigma Aldrich. Unless otherwise stated, all enzyme assays were carried out at 37°C in 50 mM Bis-Tris propane (pH 7.0) containing 5 mM MgCl_2_, 0.3 mM NADH, 1 mM phosphoenolpyruvate, 1 U lactate dehydrogenase, 1 U pyruvate kinase and 0.2 mg ml^-1^ BSA. Saturations kinetics for ADP-glucose and α-glucose 1-phosphate were measured by fixing the concentration of one substrate and whilst varying the other. The effect of pH was determined using 50 mM Na/K phosphate (pH 5.0), Bis-Tris (pH 6.0) and Bis-Tris propane (pH 7.0, 8.0 and 9.0) buffers. The dependence on pH and temperature (25–50°C) were determined using 1 mM each of ADP-glucose and glucose 1-phosphate. The activity of GlgA was also measured with rabbit liver (Sigma) and *Streptomyces venezuelae* glucan (details to be described elsewhere) as acceptors (0.3–10 mg l^-1^) with 1 mM ADP-glucose. Enzyme concentrations were such to allow reactions to progress linearly for 5 min with total donor consumption being < 10%. Initial rates (*v*_0_/[E]) were measured by monitoring the oxidation of NADH at 340 nm using a Perkin Elmer Lambda 25 spectrophotometer. One unit of enzyme activity refers to the production of 1 μmol of ADP per min. Initial rate data were fitted to the Michaelis-Menten equation without (Eq 1) or with (Eq 2) substrate inhibition using SigmaPlot version 12.3 or KaleidaGraph version 3.07, respectively.

$$\frac{v_{0}}{\left[E\right]}=\;\frac{k_{cat}\cdot \left[\text{S}\right]}{K_{m}\;+\;\left[\text{S}\right]}$$ (1)

$$\frac{v_{0}}{\left[E\right]}=\;\frac{k_{cat}\cdot \left[\text{S}\right]}{K_{m}\;+\;\left[\text{S}\right]\left(1\;+\;\frac{\left[\text{S}\right]}{K_{i}}\right)}$$ (2)

GlgA-catalyzed reactions with 1 mM each of ADP-glucose and glucose 1-phosphate were also monitored using ^1^H NMR spectroscopy. Spectra of 0.5 ml samples containing 10% v/v D_2_O at 20°C were recorded on a Bruker Avance III 400 MHz spectrometer and data were analyzed using Topspin 2.1 software spectrometer (Bruker Biospin Ltd). Other potential acceptors, including maltodextrin (dextrose equivalent 16.5–19.5; i.e. with an average DP of ~5–6), were obtained from Sigma-Aldrich. The GlgP-catalyzed reaction was also monitored using NMR spectroscopy and was assayed in either 30 mM phosphate buffer, pH 7.5, containing 1 mM pyridoxal phosphate and 30 mg ml^-1^ glycogen or in 50 mM HEPES, pH 7.5, containing 1 mM pyridoxal phosphate, 10 mM G1P and 30 mg ml^-1^ glycogen.

Finally, the products of a GlgA-catalyzed reaction were diluted 1:100 into 50% methanol/water with 0.1% NH_4_OH and infused into a Synapt G2 mass spectrometer (Waters, Manchester, UK) at 5 μl min^-1^ using a syringe pump (Harvard Apparatus, Kent, UK). The mass spectrometer was controlled by Masslynx 4.1 software (Waters), operated in resolution and negative ion mode and calibrated using sodium formate. The sample was analyzed for 2 min with 1 s MS scan time over the range of 50–1200 *m/z* with 2.5 kV capillary voltage, 40 V cone voltage and 120°C cone temperature. Leu-enkephalin peptide (2 ng ml^-1^, Waters) was infused at 10 μl min^-1^ as a lock mass and measured every 10 s. The peptide was fragmented for dual point calibration (*m/z* 236.1041 and 554.262), which was applied during the acquisition. Spectra were generated in Masslynx 4.1 by combining a number of scans, and peaks were centred using the median of areas.

### Glucan quantification in mycobacterial strains

Capsular α-glucans were determined in cell-free culture supernatants from *M*. *tuberculosis* cells grown in presence of 0.05% (v/v) tyloxapol as shaking cultures, conditions under which capsular α-glucans are stripped off and released from the cells [6]. Cultures were harvested and supernatants were filter-sterilized through a 0.22 μm pore size filter to remove remaining cells. To obtain intracellular α-glucans, *M*. *tuberculosis* cells were harvested, washed twice with PBS containing 0.05% tyloxapol to remove capsular α-glucans and media components, and subsequently extracted with hot water (95°C for 4 h).

Capsular and intracellular α-glucans were determined by sandwich ELISA as described previously [49]. Briefly, medium binding ELISA plates (Greiner) were coated with anti-α-glucan monoclonal antibody (IV58B6)[50] in a final concentration of 5.0 μg ml^-1^ in PBS and incubated overnight under gentle shaking at room temperature (RT). The plates were washed 4 times with 200 μl/well PBS containing 0.05% (v/v) Tween 20 and subsequently blocked with 200 μl/well of 1% (w/v) BSA in PBS for 1 h at RT under gentle shaking. Then, the blocking solution was removed and 100 μl/well of α-glucan samples or rabbit liver glycogen standard (twofold serial diluted in 1% (w/v) BSA in PBS) were added and incubated for 1.5 h at RT under gentle shaking. After this, the plates were washed 4 times with PBS containing 0.05% (v/v) Tween 20 before 50 μl/well of the pre-incubated secondary antibody solution (see below) was added and incubated for 1.5 h at RT under gentle shaking. Subsequently, the plates were washed 6 times with 200 μl/well PBS containing 0.05% (v/v) Tween 20 and developed by adding 100 μl/well ortho-phenylene diamine dihydrochloride (OPD) coloring solution (10 mg OPD; 10 ml OPD buffer (0.1 M citric acid; 0.2 M Na_2_HPO_4_; pH = 5.5); 5.0 μl 30% H_2_O_2_) for 4 min. The coloring was stopped by adding 50 μl/well 10% H_2_SO_4_, and optical density was measured at 492 nm using a plate reader.

The secondary antibody solution was made by first incubating 33.3 μl anti-α-glucan monoclonal antibody (1.5 mg l^-1^) with 10 μg goat-anti-mouse IgM (horse radish peroxidase-conjugate) (Life Technologies) and 56.7 μl PBS containing 0.05% (v/v) Tween 20 for 2 h at RT. Then, 100 μl clone 20 (2.0 mg l^-1^) (an irrelevant mouse IgM antibody [51]) and 50 μl normal goat serum was added and incubated for 1.5 h at RT. Finally, the pre-incubated secondary antibody solution was diluted in 5.25 ml PBS containing 1% (w/v) BSA.

Semiquantitative glucan dot blot analysis using the anti-α-glucan monoclonal antibody was done essentially as described previously [49].

For enzymatic quantifications of intracellular glucans, 25 μl aliquots of hot water cell extracts (see above) were mixed with 15 μl acetic acid (1 M), 60 μl sodium acetate (0.2 M; pH 5.2) and 5 μl amyloglucosidase solution (1000 U ml^-1^ in H_2_O, Fluka) and incubated at 57°C for 15 h. For quantification of the liberated glucose, 10 μl of this amyloglucosidase-treated sample was mixed with 100 μl glucose oxidase/peroxidase/*o*-dianisidine solution (Sigma) and incubated at 37°C for 30 min. Reactions were stopped by the addition of 75 μl sulphuric acid (6 M), and absorbance was measured at 531 nm. Controls without amyloglucosidase addition were done to correct for the presence of free glucose in the samples. Glucan concentrations were estimated using rabbit liver glycogen as a reference.

### Immunogold labeling and electron microscopy for visualization of capsular α-glucans

*M*. *tuberculosis* WT and the Δ*glgC*(u) Δ*treS* double mutant were grown in Middlebrook 7H9 liquid medium supplemented with 10% (v/v) ADC enrichment (BD, Breda, The Netherlands) and 0.05% (v/v) Tween 80 without shaking for 17 days at 37°C. Subsequently, the strains were centrifuged at 2000×*g* for 10 minutes and bacterial pellets were resuspended in PBS. The bacterial cells were fixed with 4% (w/v) paraformaldehyde and 0.4% (v/v) glutaraldehyde in PHEM buffer (120 mM PIPES, 50 mM HEPES, 20 mM EGTA, 4 mM MgCl_2_, pH 6.9) for 4 h. Next, the fixed cells were centrifuged at 2000×*g* for 10 min and carefully resuspended in storage solution (PHEM buffer containing 0.5%, w/v, paraformaldehyde). The fixed cells were spotted (3.0 μl, cell suspensions adjusted to 0.55 OD_600 nm_) onto carbon coated EM grids (EMS, Hatfield, PA, USA) and dried for 15 min at 37°C. Next, the EM grids were incubated 2 times for 2 min with 0.15% (w/v) glycine in PBS and blocked for 10 min at RT in PBS supplemented with 1% (w/v) BSA. Subsequently, the EM grids were immunolabeled for 3 h at RT using mouse anti-α-glucan monoclonal antibody at 10 μg ml^-1^ [50]. The EM grids were washed 3 times for 3 min at RT in 0.1% (w/v) BSA in PBS and incubated with biotin-conjugated goat anti-mouse IgM (0.1 μg ml^-1^, Zymed) secondary antibody in 1.0% (w/v) BSA in PBS for 45 min at 37°C. Next, the EM grids were washed 3 times for 3 min in 0.1% (w/v) BSA in PBS at RT and immunogold labeled with 10 nm-gold particle labeled goat anti-biotin tertiary antibody (1/20 dilution, Aurion) in 1.0% (w/v) BSA in PBS for 30 min at 37°C. Subsequently, EM-grids were washed 5 times for 2 min in PBS and incubated with 1% (v/v) glutaraldehyde in PBS for 5 min at RT. Finally, the EM grids were washed 6 times for 1 min with H_2_O, dried overnight and stored in a grid-box for EM analysis. The EM pictures were obtained by using Electron Microscope (CM100 BIOTRIM, Philips) and digital camera (Morada 9.2, Olympus) with ITEM imaging software (version 5.2).

### Animal infections

BALB/c mice (4 to 6-week-old females; US National Cancer Institute) were infected intravenously through the lateral tail vein with 5 × 10^4^ CFU of the indicated exponentially growing *M*. *tuberculosis* strains suspended in 200 μl PBS containing 0.05% (v/v) Tween 80. At different time points, three mice per group were killed, and bacterial burden was determined by plating serial dilutions of lung and spleen homogenates onto Middlebrook 7H10 agar plates supplemented with 10% (v/v) OADC enrichment (Becton Dickinson Microbiology Systems) and 0.5% (v/v) glycerol.

### Ethics statement

Mouse studies were performed in accordance to National Institutes of Health guidelines using recommendations in the Guide for the Care and Use of Laboratory Animals. The protocols used in this study were approved by the Institutional Animal Care and Use Committee of Albert Einstein College of Medicine (Protocol #20120114).

## Supporting Information

S1 Fig The configuration of metabolic pathways relating to α-glucans thought to exist in *M*. *tuberculosis* prior to (left panel) or after (right panel) the work currently presented. G6P, glucose 6-phosphate; G1P, glucose 1-phosphate; M1P, α-maltose 1-phosphate; T6P, trehalose 6-phosphate; ADPG, ADP-glucose; UDPG, UDP-glucose; MGLP, methylglucose lipopolysaccharide.
(TIF)

S2 Fig GlgP is a G1P-forming glycogen phosphorylase with no involvement in M1P synthesis. (A) Enzymatic activity of recombinant *M*. *tuberculosis* GlgP monitored using ^1^H NMR spectroscopy. A broad signal (~5.32 ppm) is associated with the α-1,4 linkages within the glycogen polymer in the absence of enzyme (upper ^1^H NMR spectrum). The formation of resonances consistent with G1P were clearly observed (~5.36 ppm in the lower spectrum) when GlgP was incubated with glycogen (30 mg ml^-1^) and inorganic phosphate (30 mM). There was no indication of the corresponding well-defined doublet associated with formation of M1P (~5.32 ppm). (B) Conditional silencing of the *glgE* gene in the *M*. *smegmatis* Δ*treS*(u) Δ*glgP*(u) c-*glgE*-tet-off mutant reveals no involvement of GlgP for alternative M1P synthesis. Cells were cultivated for 24 h with or without 1 μg ml^-1^ ATc as indicated, and hot water extracts from 1 ml culture aliquots (normalized to OD_600 nm_ = 0.5) were analyzed by TLC.
(TIF)

S3 Fig (Semi)quantitative detection of α-glucan in *M*. *smegmatis* mc^2^155 and *M*. *tuberculosis* H37Rv mutant strains. (A) Detection of capsular and cytosolic glucan in *M*. *smegmatis* mc^2^155 mutant strains. Cells were grown on Middlebrook 7H10 agar plates for 3 days. Extracellular (i.e. capsular) and cytosolic glucan were extracted, and aliquots of extracts were analyzed by dot blot employing an α-glucan-specific monoclonal antibody as described previously [49]. (B) Detection of cytosolic α-glucan in *M*. *tuberculosis* H37Rv mutant strains using an enzymatic method. Cells were grown in Middlebrook 7H9 liquid medium for 7 days with shaking and α-glucan from hot water cytosolic extracts was quantified using an enzymatic method. Errors represent the SEM of three experimental replicates. Values were normalized based on the OD_600 nm_ of cultures. Similar results were obtained with independent biological replicates using a sandwich ELISA method (Fig 4).
(TIF)

S4 Fig The *M*. *tuberculosis* Δ*glgC*(u) Δ*otsA* mutant is a trehalose auxotroph despite the presence of Rv3032. Trehalose auxotrophy implies that it is devoid of α-glucans usable as substrates for trehalose biosynthesis *via* the TreX-TreY-TreZ pathway. Cells were cultivated on Middlebrook 710 agar plates with or without 500 μM trehalose for 21 days.
(TIF)

S1 Table Strains of *M*. *smegmatis* mc^2^155 used in this study. Mutants were generated by allelic exchange employing specialized transduction using mycobacteriophages listed in S3 Table. Abbreviations: Kan^r^, kanamycin resistant; Hyg^r^, hygromycin resistant; Apra^r^, apramycin resistant; (u), unmarked mutant.
(PDF)

S2 Table Strains of *M*. *tuberculosis* H37Rv used in this study. Mutants were generated by allelic exchange employing specialized transduction using mycobacteriophages listed in S3 Table. Abbreviations: Hyg^r^, hygromycin resistant; (u), unmarked mutant.
(PDF)

S3 Table Oligonucleotides used for generation of allelic exchange substrates. The phages listed here were used for the generation of either gene deletion mutants of *M*. *smegmatis* mc^2^155 and *M*. *tuberculosis* H37Rv or of knock-in mutants of *M*. *smegmatis* mc^2^155 (phc-MSMEG_4916–4×tetO) listed in S1 and S2 Tables by specialized transduction.
(PDF)

S1 Text Nucleotide sequence of plasmid pcRv1327c-4XtetO. Vector arms used for generating the allelic exchange substrate to establish regulated expression of *glgE* in *M*. *smegmatis* are highlighted in grey. *Van*91I restriction sites are underlined.
(PDF)

S2 Text Nucleotide sequence of plasmid pMV261-RBS-G. The modified ribosome binding site is highlighted in grey. *Eco*RI and *Hin*dIII restriction sites used to clone *revtetR* are underlined.
(PDF)
