# Supplementary figures and images for: Metabolic Network for the Biosynthesis of Intra- and Extracellular α-Glucans Required for Virulence of Mycobacterium tuberculosis
Source: PLoS Pathog. 2016 Aug 11;12(8):e1005768. doi: 10.1371/journal.ppat.1005768 (PMC4981310; doi:10.1371/journal.ppat.1005768)

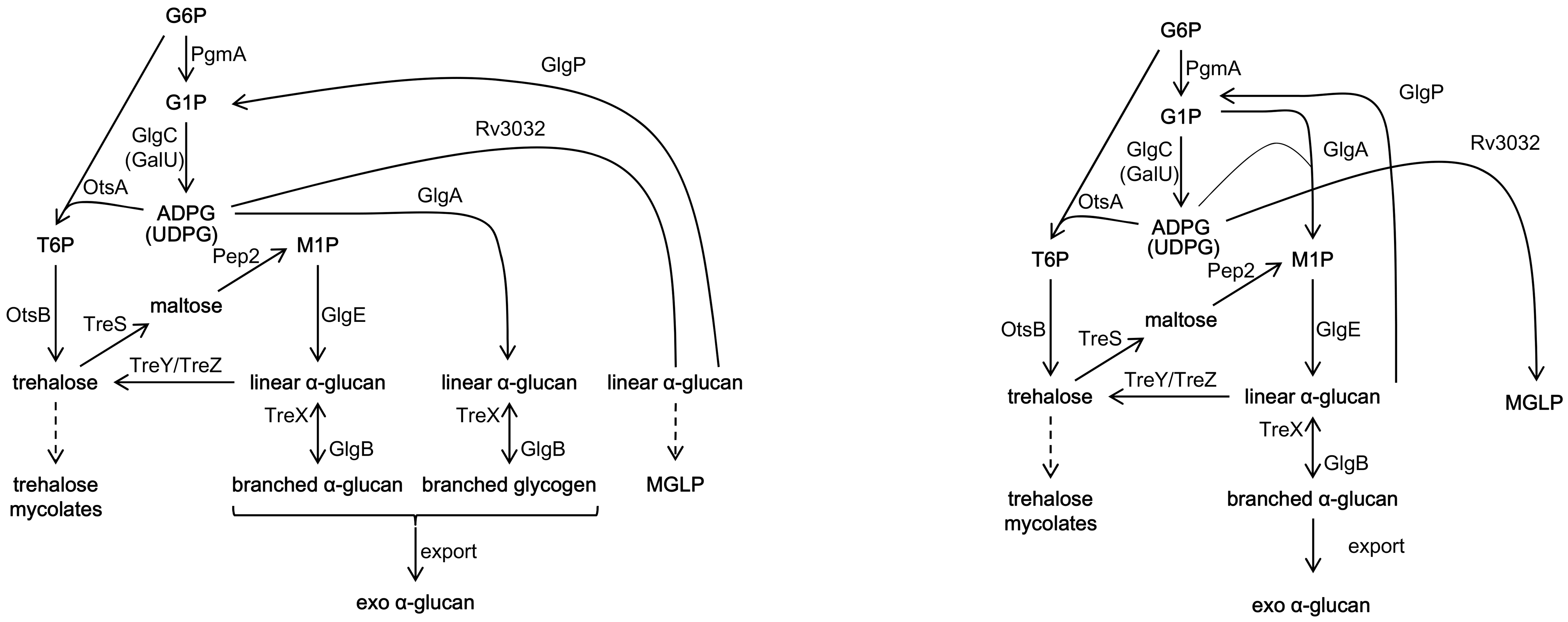

Supplement: S1 Fig — G6P, glucose 6-phosphate; G1P, glucose 1-phosphate; M1P, α-maltose 1-phosphate; T6P, trehalose 6-phosphate; ADPG, ADP-glucose; UDPG, UDP-glucose; MGLP, methylglucose lipopolysaccharide. (TIF) [file ppat.1005768.s001.tif]

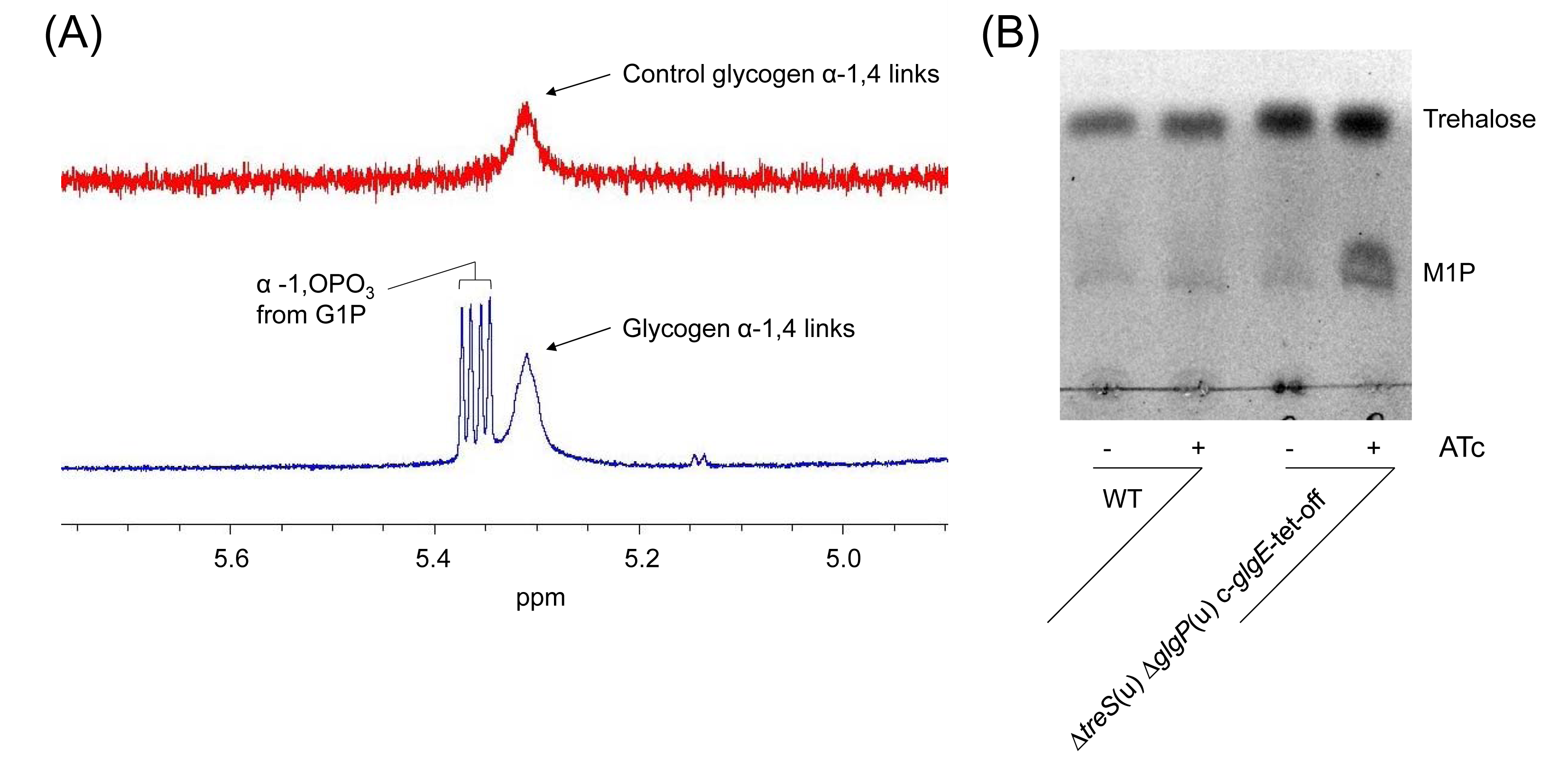

Supplement: S2 Fig — (A) Enzymatic activity of recombinant M. tuberculosis GlgP monitored using 1H NMR spectroscopy. A broad signal (~5.32 ppm) is associated with the α-1,4 linkages within the glycogen polymer in the absence of enzyme (upper 1H NMR spectrum). The formation of resonances consistent with G1P were clearly observed (~5.36 ppm in the lower spectrum) when GlgP was incubated with glycogen (30 mg ml-1) and inorganic phosphate (30 mM). There was no indication of the corresponding well-defined doublet associated with formation of M1P (~5.32 ppm). (B) Conditional silencing of the glgE gene in the M. smegmatis ΔtreS(u) ΔglgP(u) c-glgE-tet-off mutant reveals no involvement of GlgP for alternative M1P synthesis. Cells were cultivated for 24 h with or without 1 μg ml-1 ATc as indicated, and hot water extracts from 1 ml culture aliquots (normalized to OD600 nm = 0.5) were analyzed by TLC. (TIF) [file ppat.1005768.s002.tif]

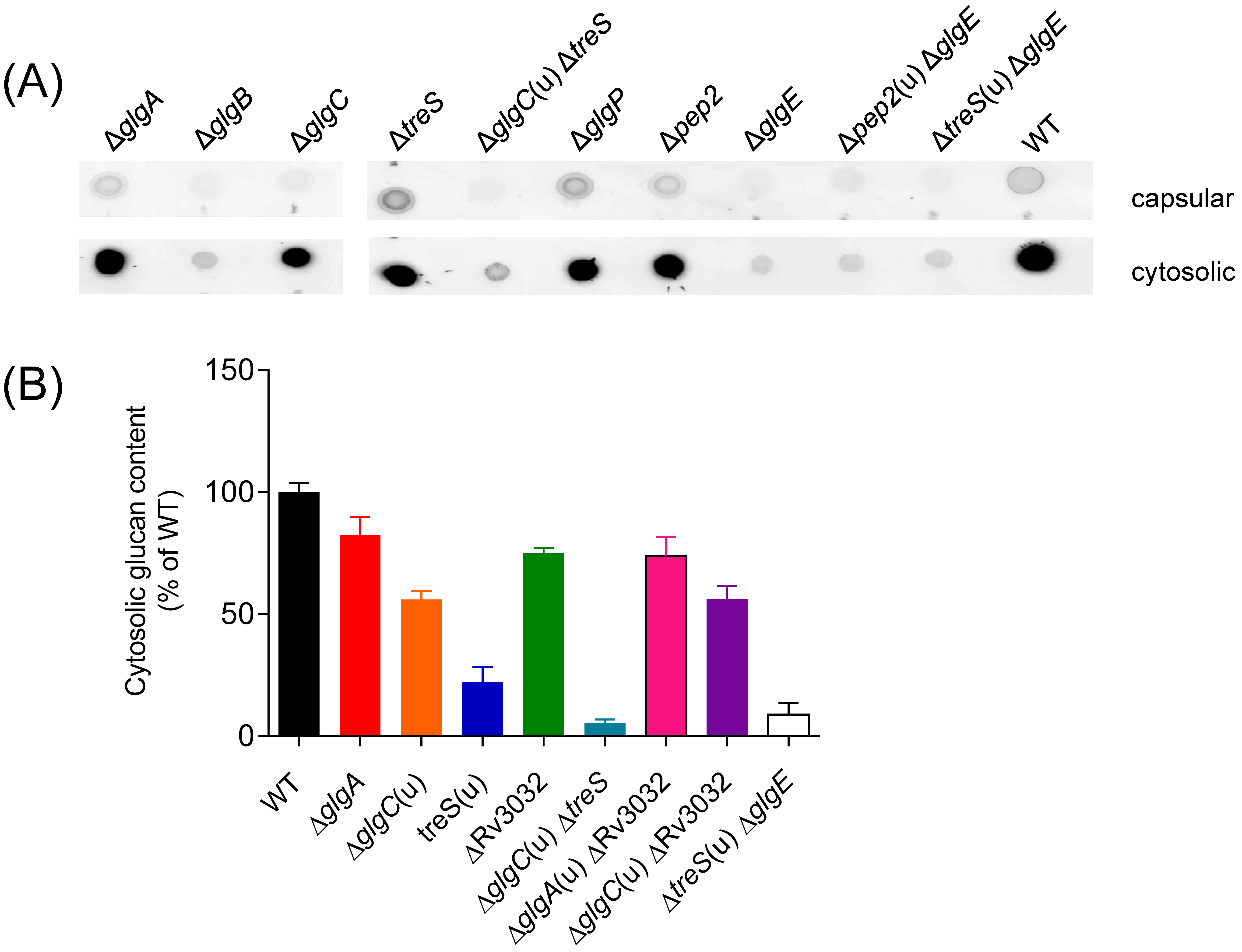

Supplement: S3 Fig — (A) Detection of capsular and cytosolic glucan in M. smegmatis mc2155 mutant strains. Cells were grown on Middlebrook 7H10 agar plates for 3 days. Extracellular (i.e. capsular) and cytosolic glucan were extracted, and aliquots of extracts were analyzed by dot blot employing an α-glucan-specific monoclonal antibody as described previously [49]. (B) Detection of cytosolic α-glucan in M. tuberculosis H37Rv mutant strains using an enzymatic method. Cells were grown in Middlebrook 7H9 liquid medium for 7 days with shaking and α-glucan from hot water cytosolic extracts was quantified using an enzymatic method. Errors represent the SEM of three experimental replicates. Values were normalized based on the OD600 nm of cultures. Similar results were obtained with independent biological replicates using a sandwich ELISA method (Fig 4). (TIF) [file ppat.1005768.s003.tif]

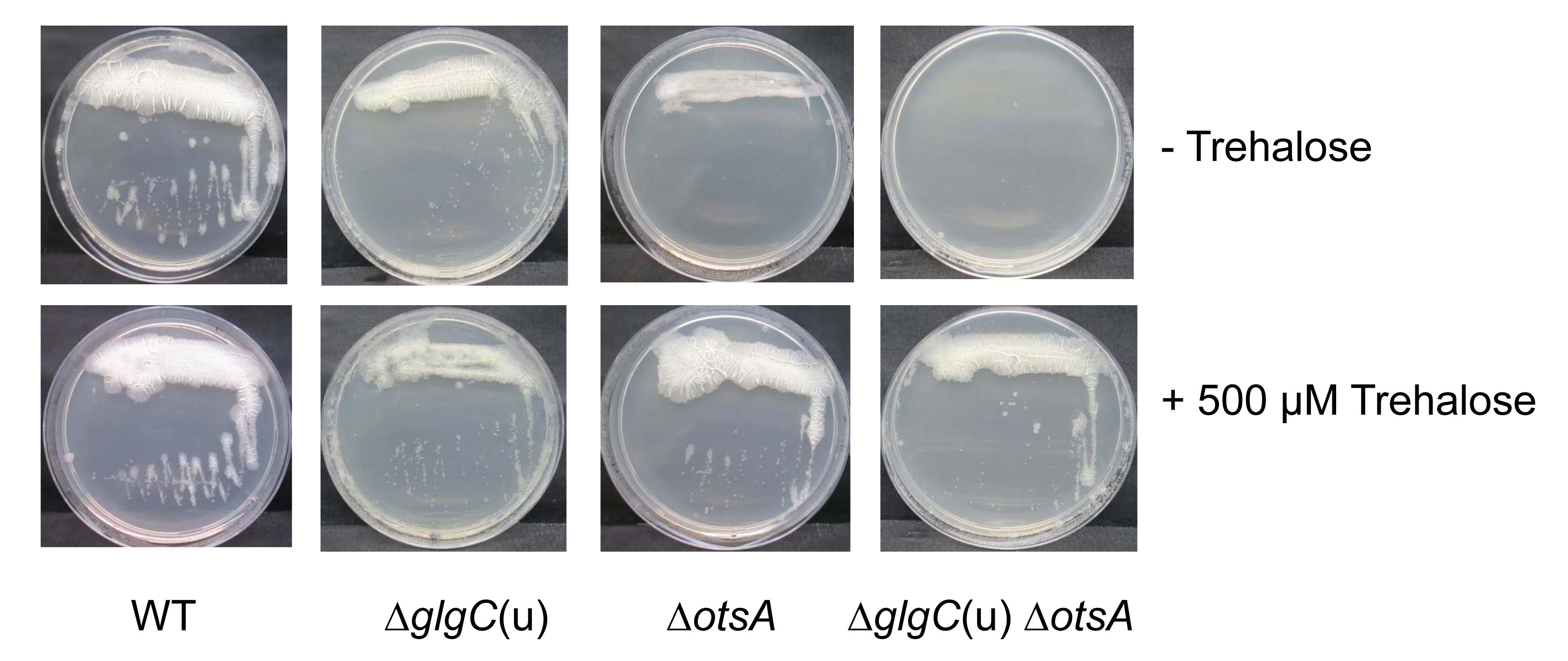

Supplement: S4 Fig — Trehalose auxotrophy implies that it is devoid of α-glucans usable as substrates for trehalose biosynthesis via the TreX-TreY-TreZ pathway. Cells were cultivated on Middlebrook 710 agar plates with or without 500 μM trehalose for 21 days. (TIF) [file ppat.1005768.s004.tif]
