# Supplementary material for: Metabolic Network for the Biosynthesis of Intra- and Extracellular α-Glucans Required for Virulence of Mycobacterium tuberculosis
Source: PLoS Pathog. 2016 Aug 11;12(8):e1005768. doi: 10.1371/journal.ppat.1005768 (PMC4981310; doi:10.1371/journal.ppat.1005768)
Supplement: S1 Table — Mutants were generated by allelic exchange employing specialized transduction using mycobacteriophages listed in S3 Table. Abbreviations: Kanr, kanamycin resistant; Hygr, hygromycin resistant; Aprar, apramycin resistant; (u), unmarked mutant. (PDF) [file ppat.1005768.s005.pdf]

S1 Table. Strains of *M. smegmatis* mc^{2}155 used in this study. Mutants were generated by allelic exchange employing specialized transduction using mycobacteriophages listed in **S3 Table**. Abbreviations: Kan^r, kanamycin resistant; Hyg^r, hygromycin resistant; Apra^r, apramycin resistant; (u), unmarked mutant.

Strain	Plasmid(s)	Relevant characteristics	Source or reference
Δ*glgA*		Δ*glgA*::γδ*res-sacB-hyg*-γδ*res*; Hyg^r	this study
Δ*glgA*(u)		Δ*glgA*::γδ*res*	this study
Δ*glgB*		Δ*glgB*::γδ*res-sacB-hyg*-γδ*res*; Hyg^r	[15]
Δ*glgC*		Δ*glgC*::γδ*res-sacB-hyg*-γδ*res*; Hyg^r	this study
Δ*glgC*(u)		Δ*glgC*::γδ*res*	this study
Δ*glgE*		Δ*glgE*::γδ*res-sacB-hyg*-γδ*res*; Hyg^r	[15]
Δ*pep2*		Δ*pep2*::γδ*res-sacB-hyg*-γδ*res*; Hyg^r	[15]
Δ*pep2*(u)		Δ*pep2*::γδ*res*	this study
Δ*treS*(u)		Δ*treS*::γδ*res*	[15]
Δ*glgA*(u) Δ*glgB*		Δ*glgA*::γδ*res* Δ*glgB*::γδ*res-sacB-hyg*-γδ*res*; Hyg^r	this study
Δ*glgA*(u) Δ*treS*		Δ*glgA*::γδ*res* Δ*treS*::γδ*res-sacB-hyg*-γδ*res*; Hyg^r	this study
Δ*glgA*(u) Δ*treS*(u)		Δ*glgA*::γδ*res* Δ*treS*::γδ*res*	this study
Δ*glgC*(u) Δ*glgB*		Δ*glgC*::γδ*res* Δ*glgB*::γδ*res-sacB-hyg*-γδ*res*; Hyg^r	this study
Δ*glgC*(u) Δ*treS*		Δ*glgC*::γδ*res* Δ*treS*::γδ*res-sacB-hyg*-γδ*res*; Hyg^r	this study
Δ*glgC*(u) Δ*treS*(u)		Δ*glgC*::γδ*res* Δ*treS*::γδ*res*	this study
Δ*glgA*(u) Δ*otsA*		Δ*glgA*::γδ*res* Δ*otsA*::γδ*res-sacB-hyg*-γδ*res*; Hyg^r	this study
Δ*pep2*(u) Δ*glgE*		Δ*pep2*::γδ*res* Δ*glgE*::γδ*res-sacB-hyg*-γδ*res*; Hyg^r	this study
Δ*treS*(u) Δ*glgB*		Δ*treS*::γδ*res* Δ*glgB*::γδ*res-sacB-hyg*-γδ*res*; Hyg^r	this study
Δ*treS*(u) Δ*glgE*		Δ*treS*::γδ*res* Δ*glgE*::γδ*res-sacB-hyg*-γδ*res*; Hyg^r	this study
Δ*treS*(u) Δ*glgP*		Δ*treS*::γδ*res* Δ*glgP*::γδ*res-sacB-hyg*-γδ*res*; Hyg^r	this study
Δ*treS*(u) Δ*glgP*(u)		Δ*treS*::γδ*res* Δ*glgP*::γδ*res*	this study
Δ*glgA*(u) Δ*treS*(u) Δ*glgB*		Δ*glgA*::γδ*res* Δ*treS*::γδ*res* Δ*glgB*::γδ*res-sacB-hyg*-γδ*res*; Hyg^r	this study
Δ*glgC*(u) Δ*treS*(u) Δ*glgB*		Δ*glgC*::γδ*res* Δ*treS*::γδ*res* Δ*glgB*::γδ*res-sacB-hyg*-γδ*res*; Hyg^r	this study
Δ*glgE*(u) Δ*pep2 glgC:IS1096*		Δ*glgE*::γδ*res* Δ*pep2*::γδ*res-sacB-hyg*-γδ*res*; *IS1096* insertion 10 bp upstream of *glgC* start codon; Hyg^r	[15]
Δ*glgE*(u) Δ*pep2 glgC:IS1096*	pMV361::Rv1213	Δ*glgE*::γδ*res* Δ*pep2*::γδ*res-sacB-hyg*-γδ*res*; *IS1096* insertion 10 bp upstream of *glgC* start codon; constitutive expression of *M. tuberculosis glgC* (Rv1213) from *groEL2* (hsp60) promoter on integrative plasmid pMV361; Hyg^r, Kan^r	this study

continued…

S1 Table continued

Strain	Plasmid(s)	Relevant characteristics	Source or reference
c-*glgE*-4×*tetO*		Knock-in mutant harboring *hyg*-P*myc1*-4×*tetO* cassette upstream of *glgE* start codon; Hyg^r	this study
c-*glgE*-4×*tetO*	pMV261::revtetR_RBS-G	Conditional mutant harboring *hyg*-P*myc1*-4×*tetO* cassette upstream of *glgE* start codon; constitutive expression of mutated *E. coli* Tn10 *tetR* (revtetR) from *groEL2* (hsp60) promoter on episomal plasmid pMV261; Hyg^r, Kan^r	this study
Δ*treS*(u) c-*glgE*-4×*tetO*		Δ*treS*::γδ*res*; knock-in mutant harboring *hyg*-P*myc1*-4×*tetO* cassette upstream of *glgE* start codon; Hyg^r	this study
Δ*treS*(u) c-*glgE*-4×*tetO*	pMV261::revtetR_RBS-G	Δ*treS*::γδ*res*; conditional mutant harboring *hyg*-P*myc1*-4×*tetO* cassette upstream of *glgE* start codon; constitutive expression of mutated *E. coli* Tn10 *tetR* (revtetR) from *groEL2* (hsp60) promoter on episomal plasmid pMV261; Hyg^r, Kan^r	this study
Δ*treS*(u) Δ*glgA*(u) c-*glgE*-4×*tetO*		Δ*treS*::γδ*res* Δ*glgA*::γδ*res*; knock-in mutant harboring *hyg*-P*myc1*-4×*tetO* cassette upstream of *glgE* start codon; Hyg^r	this study
Δ*treS*(u) Δ*glgA*(u) c-*glgE*-4×*tetO*	pMV261::revtetR_RBS-G	Δ*treS*::γδ*res* Δ*glgA*::γδ*res*; conditional mutant harboring *hyg*-P*myc1*-4×*tetO* cassette upstream of *glgE* start codon; constitutive expression of mutated *E. coli* Tn10 *tetR* (revtetR) from *groEL2* (hsp60) promoter on episomal plasmid pMV261; Hyg^r, Kan^r	this study
Δ*treS*(u) Δ*glgA*(u) c-*glgE*-4×*tetO*	pMV261::revtetR_RBS-G pMV361(Apra)	Δ*treS*::γδ*res* Δ*glgA*::γδ*res*; conditional mutant harboring *hyg*-P*myc1*-4×*tetO* cassette upstream of *glgE* start codon; constitutive expression of mutated *E. coli* Tn10 *tetR* (revtetR) from *groEL2* (hsp60) promoter on episomal plasmid pMV261; carrying empty integrative plasmid pMV361;Hyg^r, Kan^r, Apra^r	this study
Δ*treS*(u) Δ*glgA*(u) c-*glgE*-4×*tetO*	pMV261::revtetR_RBS-G pMV361(Apra)::Rv1212c	Δ*treS*::γδ*res* Δ*glgA*::γδ*res*; conditional mutant harboring *hyg*-P*myc1*-4×*tetO* cassette upstream of *glgE* start codon; constitutive expression of mutated *E. coli* Tn10 *tetR* (revtetR) from *groEL2* (hsp60) promoter on episomal plasmid pMV261; constitutive expression of *M. tuberculosis* Rv1212c (*glgA*) from *groEL2* (hsp60) promoter on integrative plasmid pMV361;Hyg^r, Kan^r, Apra^r	this study
Δ*treS*(u) Δ*glgC*(u) c-*glgE*-4×*tetO*		Δ*treS*::γδ*res* Δ*glgC*::γδ*res*; knock-in mutant harboring *hyg*-P*myc1*-4×*tetO* cassette upstream of *glgE* start codon; Hyg^r	this study
Δ*treS*(u) Δ*glgC*(u) c-*glgE*-4×*tetO*	pMV261::revtetR_RBS-G	Δ*treS*::γδ*res* Δ*glgC*::γδ*res*; conditional mutant harboring *hyg*-P*myc1*-4×*tetO* cassette upstream of *glgE* start codon; constitutive expression of mutated *E. coli* Tn10 *tetR* (revtetR) from *groEL2* (hsp60) promoter on episomal plasmid pMV261; Hyg^r, Kan^r	this study
Δ*treS*(u) Δ*glgP*(u) c-*glgE*-4×*tetO*		Δ*treS*::γδ*res* Δ*glgP*::γδ*res*; knock-in mutant harboring *hyg*-P*myc1*-4×*tetO* cassette upstream of *glgE* start codon; Hyg^r	this study
Δ*treS*(u) Δ*glgP*(u) c-*glgE*-4×*tetO*	pMV261::revtetR_RBS-G	Δ*treS*::γδ*res* Δ*glgP*::γδ*res*; conditional mutant harboring *hyg*-P*myc1*-4×*tetO* cassette upstream of *glgE* start codon; constitutive expression of mutated *E. coli* Tn10 *tetR* (revtetR) from *groEL2* (hsp60) promoter on episomal plasmid pMV261; Hyg^r, Kan^r	this study
