# Supplementary material for: Metabolic Network for the Biosynthesis of Intra- and Extracellular α-Glucans Required for Virulence of Mycobacterium tuberculosis
Source: PLoS Pathog. 2016 Aug 11;12(8):e1005768. doi: 10.1371/journal.ppat.1005768 (PMC4981310; doi:10.1371/journal.ppat.1005768)
Supplement: S2 Table — Mutants were generated by allelic exchange employing specialized transduction using mycobacteriophages listed in S3 Table. Abbreviations: Hygr, hygromycin resistant; (u), unmarked mutant. (PDF) [file ppat.1005768.s006.pdf]

S2 Table. Strains of *M. tuberculosis* H37Rv used in this study. Mutants were generated by allelic exchange employing specialized transduction using mycobacteriophages listed in **S3 Table**. Abbreviations: Hyg^r, hygromycin resistant; (u), unmarked mutant.

Strain	Relevant characteristics	Source or reference
Δ*glgA*	Δ*glgA*::γδ*res-sacB-hyg*-γδ*res*; Hyg^r	this study
Δ*glgA*(u)	Δ*glgA*::γδ*res*	this study
Δ*glgC*(u)	Δ*glgC*::γδ*res*	[15]
Δ*otsA*	Δ*otsA*::γδ*res-sacB-hyg*-γδ*res*; Hyg^r	this study
Δ*treS*(u)	Δ*treS*::γδ*res*	[15]
ΔRv3032	ΔRv3032::γδ*res-sacB-hyg*-γδ*res*; Hyg^r	[15]
Δ*glgA*(u) ΔRv3032	Δ*glgA*::γδ*res* ΔRv3032::γδ*res-sacB-hyg*-γδ*res*; Hyg^r	this study
Δ*glgC*(u) Δ*otsA*	Δ*glgC*::γδ*res* Δ*otsA*::γδ*res-sacB-hyg*-γδ*res*; Hyg^r	this study
Δ*glgC*(u) Δ*treS*	Δ*glgC*::γδ*res* Δ*treS*::γδ*res-sacB-hyg*-γδ*res*; Hyg^r	[15]
Δ*glgC*(u) ΔRv3032	Δ*glgC*::γδ*res* ΔRv3032::γδ*res-sacB-hyg*-γδ*res*; Hyg^r	[15]
Δ*treS*(u) Δ*glgE*	Δ*treS*::γδ*res* Δ*glgE*::γδ*res-sacB-hyg*-γδ*res*; Hyg^r	[15]
