# Supplementary material for: Metabolic Network for the Biosynthesis of Intra- and Extracellular α-Glucans Required for Virulence of Mycobacterium tuberculosis
Source: PLoS Pathog. 2016 Aug 11;12(8):e1005768. doi: 10.1371/journal.ppat.1005768 (PMC4981310; doi:10.1371/journal.ppat.1005768)
Supplement: S3 Table — The phages listed here were used for the generation of either gene deletion mutants of M. smegmatis mc2155 and M. tuberculosis H37Rv or of knock-in mutants of M. smegmatis mc2155 (phc-MSMEG_4916–4×tetO) listed in S1 and S2 Tables by specialized transduction. (PDF) [file ppat.1005768.s007.pdf]

S3 Table. Oligonucleotides used for generation of allelic exchange substrates. The phages listed here were used for the generation of either gene deletion mutants of *M. smegmatis* mc^{2}155 and *M. tuberculosis* H37Rv or of knock-in mutants of *M. smegmatis* mc^{2}155 (phc-MSMEG_4916-4×tetO) listed in **S1 + S2 Tables** by specialized transduction.

	**Upstream flanking sequence**			**Downstream flanking sequence**			**Resulting Phage**
	**5` primer**	**3` primer**	**Restriction site**	**5` primer**	**3` primer**	**Restriction site**	
***M. smegmatis* mc^{2}155**							
Δ*glgA*	5´ TTTTTCCATAAATTGGCGATGT-GGAACTGCATCATCTG 3'	5' TTTTTCCATTTCTTGGCGCATAT-CTGGAACGGTAATGG 3'	*Van*91I	5' TTTTTCCATAGATTGGTGTCTGC-CTGAGCCGAATCC 3'	5' TTTTTCCATCTTTTGGCTCGACT-ACCACGACACCGAATG 3'	*Van*91I	phMSMEG_5080S
Δ*glgB*	5' TTTTTTTTGCATAAATTGCCTTG-GCGAAACTCGGCTTCAC 3'	5' TTTTTTTTGCATTTCTTGCTCAT-CTCGCCCTCATTCCCTG 3'	*Bst*API	5' TTTTTTTTCCATAGATTGGCGCT-GTCGATGCTCTGGTTC 3'	5' TTTTTTTTCCATCTTTTGGCGC-GCAGATGTTCGGTATCC 3'	*Van*91I	phMSMEG_4918S
Δ*glgC*	5' TTTTTCCATAAATTGGGACCCAC-GAGGACACTTGGTAAC 3'	5' TTTTTCCATTTCTTGGCTCATGG-CTCAAACCTATCCGTCTG 3'	*Van*91I	5' TTTTTCCATAGATTGGTCGGCGT-CGATCTCGACAAG 3'	5' TTTTTCCATCTTTTGGCTACGAC-GAGTACCGGCAGAAG 3'	*Van*91I	phMSMEG_5078S
Δ*glgE*	5' TTTTTTTTGCATAAATTGCGATC-CGATGCGGACTTGAGGTG 3'	5' TTTTTTTTGCATTTCTTGCCTGT-GGTTTGACCCGTTTGCC 3'	*Bst*API	5' TTTTTTTTCCATAGATTGGTGTG-CTGAACATGCCACTCATAC 3'	5' TTTTTTTTCCATCTTTTGGTGC-TCATCGGCTCGAAGACTG 3'	*Van*91I	phMSMEG_4916S
Δ*otsA*	5' TTTTTCAGAAACTGTCCCGACGA-AACAGTCAGACAAC 3'	5' TTTTTCAGTTCCTGACCTTC-GTAGTACTTCGCGACATC 3'	*Alw*NI	5' TTTTTCCATAGATTGGCGGTCAA-CCAGAACCCAGAAGAG 3'	5' TTTTTCCATCTTTTGGTGGAAAT-CGCAACCGGCAAAG 3'	*Van*91I	phMSMEG_5892S
Δ*glgP*	5' TTTTTGCATAAATTGCTCTGTGG-TTTGACCCGTTTGC 3'	5' TTTTTGCATTTCTTGCGAGAAGT-ACGCGATGCCCTTG 3'	*Bst*API	5' TTTTTCCATAGATTGGCAATGGG-ACCGACGTGTTCTC 3'	5' TTTTTCCATCTTTTGGCAGTTCG-GCACGCTCAGTTC 3'	*Van*91I	phMSMEG_4915S
Δ*treS*	5' TTTTTCCATAAATTGGGTGTTTA-GCCCGCGGAGAATC 3'	5' TTTTTCCATTTCTTGGCGTGCTT-GAACCAGTTGGTGTC 3'	*Van*91I	5' TTTTTCCATAGATTGGGTACGGC-TACCACTCTGTGAACG 3'	5' TTTTTCCATCTTTTGGGACCCG-GTTGAGTTCGATGTC 3'	*Van*91I	phMSMEG_6515S
c-*glgE*	5' TTTTTCCATAAATTGGGATCCGA-TGCGGACTTGAGGTGG 3'	5' TTTTTCCATTTCTTGGACCGACG-ACGGCTTTGGCGGGG 3'	*Van*91I	5' TTTTTGCATAGATTGCATGAGGA-GTGGTTGGGTGGCCGG 3'	5' TTTTTGCATCTTTTGCGCGGCCT-CCAGCAGGGGGTCGCG 3'	*Bst*API	phc-MSMEG_4916-4xtetO
***M. tuberculosis* H37Rv**							
Δ*glgA*	5` TTTTTCCATAAATTGGGGCGGTC-CAGTGAATGCGACTTG 3`	5` TTTTTCCATTTCTTGGGGTTCGA-GCGAATGTGCGGTC 3`	*Van*91I	5` TTTTTCCATAGATTGGGGTAACA-GCCGCGCACCGATTCAG 3`	5` TTTTTCCATCTTTTGGCCGCCGC-ACTGGGTGACGAACTC 3`	*Van*91I	phRv1212cS
Δ*otsA*	5` TTTTTCCATAAATTGGGCGTGGC-TGACCCAAGAACT 3`	5` TTTTTCCATTTCTTGGTCGATTG-GCTACCACCACGA 3`	*Van*91I	5` TTTTTGCATAGATTGCCTGGGCA-CAGTCGTTTCTCG 3`	5` TTTTGCATCTTTTGCCACCTGGA-AGGTCCACAGCA 3`	*BstAP*I	phRv3490S
ΔRv3032	5' TTTTTGCATAAATTGCGCCTTCG-ACACCGAGTTGTTC 3'	5' TTTTTGCATTTCTTGCACAACAC-GACGACATCGTGAC 3'	*Bst*API	5' TTTTTCCATAGATTGGGTGAAGC-GGTCATCAATGGACAG 3'	5' TTTTTCCATCTTTTGGTGTCAA-ACGGCGTCTTCGATAC 3'	*Van*91I	phRv3032S
