# Supplementary material for: Metabolic Network for the Biosynthesis of Intra- and Extracellular α-Glucans Required for Virulence of Mycobacterium tuberculosis
Source: PLoS Pathog. 2016 Aug 11;12(8):e1005768. doi: 10.1371/journal.ppat.1005768 (PMC4981310; doi:10.1371/journal.ppat.1005768)
Supplement: S1 Text — Vector arms used for generating the allelic exchange substrate to establish regulated expression of glgE in M. smegmatis are highlighted in grey. Van91I restriction sites are underlined. (PDF) [file ppat.1005768.s008.pdf]

S1 Text. Nucleotide sequence of plasmid pcRv1327c-4XtetO. Vector arms used for generating the allelic exchange substrate to establish regulated expression of *glgE* in *M. smegmatis* are highlighted in grey. *Van*91I restriction sites are underlined.

```
   1 acgttttgca gcagcagtcg cttcacgttc gctcgcgtat cggtgattca ttctgctaac cagtaaggca accccgccag
  81 cctagccggg tcctcaacga caggagcacg atcatgcgca cccgtcagat ccagacatga taagatacat tgatgagttt
 161 ggacaaacca caactagaat gcagtgaaaa aaatgcttta tttgtgaaat ttgtgatgct attgctttat ttgtaaccat
 241 tataagctgc aataaacaag ttcgcgaatt gataattatt atcatttgcg ggtcctttcc ggcgatccgc cttgttacgg
 321 ggcggcgacc tcgcgggttt tcgctattta tgaaaatttt ccggtttaag gcgtttccgt tcttcttcgt cataacttaa
 401 tgtttttatt taaaataccc tctgaaaaga aaggaaacga caggtgctga aagcgagctt tttggcctct gtcgtttcct
 481 ttctctgttt ttgtccgtgg aatgaacaat ggaagtcaac aaaaagcaag ctagcttatc gatgataagc ggtcaaacat
 561 gagaattcgc ggccgcataa tacgactcac tatagggatc ttaattaagg cgcctgatca ggatcgtaat acgactcact
 641 ccaaaaattg gggccaggat ctcttgtttg atgcgatgtt cctggtcgcc accgtagagg cggtcggtga cgttgcgcag
 721 gtcgtgctcg ttctccggga tatcagaatc caacaagagc aacggaaccc tacccacctg cgctacccag atccgggcgc
 801 gcaacacggc gttgtctccc agggcgacct cgaccagcac tggatccccg ttggcgtcgg tgagcagacg caacggcagc
 881 ccttgcgggt ccagcgatgg gtaggtctcg tgctgccagc cgtccgcggt aagcgattgc cggaagtagc cggagcggta
 961 gtacaacccc accgcgatca gcggcacgcc cagatcggac gcggatttca gatggtcgcc ggcgagaatc ccaagaccgc
1041 ccgagtaatt aggcaacacc tcggctaccc cgaactccag cgagaagtac gcgatcccgg tcggcagggc ttgtgcggct
1121 accccggcgt cctgctgctc ctgataccac agcggacggc tcaggtagtc gttcaagtcg gccgccagct catcgagggc
1201 gcccaaaaat tctgcgtcca gcgcaagttc gtcgagacgc gctgggttca ccgcgcccag cagcgccacc ggatcatgac
1281 cgcattgctc ccacagtgca gggtcgatcg ccgcgaacag atcctgtgtc ggtttgtccc aggaccaccg cagattggta
1361 gacagctggt ccagggcggc aagacgttcg ggtaggtggg ctcggacggt aaaccggcga agggctttca cgcgtcctta
1441 ccttactgag gatgccaccg cgtgcagtgc gcccgccgac cgctgtgcct tcgcgggtgt gatcggatac tagggtgggt
1521 atcgggcgca agaaggacac acaaggaacg acacatacaa gatgtcccgc caagaaatgg gagcatgaga ttatcaaaaa
1601 ggatcttcac ctagatcctt ttaaattaaa aatgaagttt taaatcaatc taaagtatat atgagtaaac ttggtctgac
1681 agttaccaat gcttaatcag tgaggcacct atctcagcga tctgtctatt tcgttcatcc atagttgcct gactccccga
1761 cgtggccgac cagcccgtca tcgtcaacgc ctgaccgcgg tgcggacagg ccgtgtcgcg accggccgtg cggaattaag
1841 ccggcccgta ccctgtgaat agaggtccgc tgtgacacaa gaatccctgt tacttctcga ccgtattgat tcggatgatt
1921 cctacgcgag cctgcggaac gaccaggaat tctgggagcc gctggcccgc cgagccctgg aggagctcgg gctgccggtg
2001 ccgccggtgc tgcgggtgcc cggcgagagc accaaccccg tactggtcgg cgagcccgac ccggtgatca agctgttcgg
2081 cgagcactgg tgcggtccgg agagcctcgc gtcggagtcg gaggcgtacg cggtcctggc ggacgccccg gtgccggtgc
2161 cccgcctcct cggccgcggc gagctgcggc ccggcaccgg agcctggccg tggccctacc tggtgatgag ccggatgacc
2241 ggcaccacct ggcggtccgc gatggacggc acgaccgacc ggaacgcgct gctcgccctg gcccgcgaac tcggccgggt
2321 gctcggccgg ctgcacaggg tgccgctgac cgggaacacc gtgctcaccc cccattccga ggtcttcccg gaactgctgc
2401 gggaacgccg cgcggcgacc gtcgaggacc accgcgggtg gggctacctc tcgccccggc tgctggaccg cctggaggac
2481 tggctgccgg acgtggacac gctgctggcc ggccgcgaac cccggttcgt ccacggcgac ctgcacggga ccaacatctt
2561 cgtggacctg gccgcgaccg aggtcaccgg gatcgtcgac ttcaccgacg tctatgcggg agactcccgc tacagcctgg
2641 tgcaactgca tctcaacgcc ttccggggcg accgcgagat cctggccgcg ctgctcgacg gggcgcagtg gaagcggacc
2721 gaggacttcg cccgcgaact gctcgccttc accttcctgc acgacttcga ggtgttcgag gagaccccgc tggatctctc
2801 cggcttcacc gatccggagg aactggcgca gttcctctgg gggccgccgg acaccgcccc cggcgcctga ggatccctag
2881 ctctagaaat attggatcgt cggcaccgtc acggccgtgg gaggcggcac gatccgcgac gtgatgatcg gccgcatccc
2961 cacggtgctg cgcagtgagc tctacgccat cccggtccct atcagtgata gagaacgcac atccctatca gtgatagaga
3041 ctctgacgag cgggagatcc ctatcagtga tagagagttt gtcctcccta tcagtgatag ataggctctg ggagtacccg
3121 tgtgtacgac cagcacggca tacatcattt cgacgccgag agattcgccg cccgaaatga gcacgatccg cattcgaagc
3201 taaggaggcc atagattgga tgagtggccg ggcaatcgga acggaaacgg agtggtgggt gcccggtcgt gtcgaaatcg
3281 atgacgtcgc gcccgtcgtt tcgtgcggcg tatatcccgc caaggcggtg gtcggcgagg tggtcccggt cagcgcggcg
3361 gtctggcgtg aaggccacga ggccgtcgca gcgacgctgg tcgtgcgcta cctcggagtg cgttacccac acctcaccga
3441 cagaccccgg gccagggtgc ttccgacgcc gagcgagccc caacaacgcg tcaagccgct gctgatcccg atgacgagcg
3521 gccaggagcc cttcgttttc cacggccagt tcacccccga ccgggtcgga ttgtggacct tccgggtgga tggttggggt
3601 gacccgatcc acacctggcg ccatgggctg atagccaagc tagatgccgg ccagggagag accgagctgt ccaacgacct
3681 gttggtaggc gcggtgctgt tggagcgcgc ggcgaccggt gtgccgcgcg ggttacgcga tcccctcctg gcggccgcgg
3761 cagcgctgcg gacccccggt gacccggtga cccgcaccgc gttggccctg acaccggaaa tcgaagagct gctggccgac
3841 tatccgctgc gggacctggt cacccggggc gagcaattcg gcgtctgggt ggatcggccg ttggcccggt tcggcgcttg
3921 gtatgagatg tttccgcgct caaccggcgg gtgggacgac gacggcaacc cggtacacgg caccttcgcc accgctgcgg
4001 cagaacttcc gcgcatcgcc ggcatggggt tcgacgtggt gtacctgccg ccgatccatc caattggcaa ggtgcatcgc
4081 aagggtcgca acaactcgcc cccaaaagtt ggcaggtttg acagcttatc atcgcgataa gctcatgacc aaaatccctt
4161 aacgtgagtt ttcgttcgac tgagcgtcag accccgtaga aaatatcaaa ggatcttctt gagatccttt ttttctgcgc
4241 gtaatctgct gcttgcaaac aaaaaaacca ccgctaccag cggtggtttg tttgccggat caagagctac caactctttt
4321 tccgaaggta actggcttca gcagagcgca gataccaaat actgtccttc tagtgtagcc gtagttaggc caccacttca
4401 agaactctgt agcaccgcct acatacctcg ctctgctaat cctgttacca gtggctgctg ccagtggcga taagtcgtgt
4481 cttaccgggt tggactcaag acgatagtta ccggataagg cgcagcggtc gggctgaacg gggggttcgt gcacacagcc
4561 cagcttggag cgaacgacct acaccgaact gagataccta cagcgtgagc tatgagaaag cgccacgctt cccgaaggga
4641 gaaaggcgga caggtatccg gtaagcggca gggtcggaac aggagagcgc acgagggagc ttccaggggg aaacgcctgg
4721 tatctttata gtcctgtcgg gtttcgccac ctctgacttg agcgtcgatt tttgtgatgc tcgtcagggg ggcggagcct
4801 atggaaaaac gccagcaacg cggccttttt acggttcctg gccttttgct ggccttttgc tggccttttg ctcacatgtt
4881 ctttcctgcg ttatcccctg attctgtgga taaccgtatt accgcctttg agtgagctga taccgctcgc cgcagccgaa
4961 cgaccgagcg cagcgagtca gtgagcgagg aagcggaaga gcgctgactt ccgcgtttcc agactttacg aaacacggaa
5041 accgaagacc attcatgttg ttgctcaggt cgcag
```
