# Supplementary material for: Metabolic Network for the Biosynthesis of Intra- and Extracellular α-Glucans Required for Virulence of Mycobacterium tuberculosis
Source: PLoS Pathog. 2016 Aug 11;12(8):e1005768. doi: 10.1371/journal.ppat.1005768 (PMC4981310; doi:10.1371/journal.ppat.1005768)
Supplement: S2 Text — The modified ribosome binding site is highlighted in grey. EcoRI and HindIII restriction sites used to clone revtetR are underlined. (PDF) [file ppat.1005768.s009.pdf]

S2 Text. Nucleotide sequence of plasmid pMV261-RBS-G. The modified ribosome binding site is highlighted in grey. *Eco*RI and *Hin*dIII restriction sites used to clone *revtetR* are underlined.

```
   1 gctagccaac aaagcgacgt tgtgtctcaa aatctctgat gttacattgc acaagataaa aatatatcat catgaacaat
  81 aaaactgtct gcttacataa acagtaatac aaggggtgtt atgagccata ttcaacggga aacgtcttgc tcgaggccgc
 161 gattaaattc caacatggat gctgatttat atgggtataa atgggctcgc gataatgtcg ggcaatcagg tgcgacaatc
 241 tatcgcttgt atgggaagcc ccatgcgcca gagttgtttc tgaaacatgg caaaggtagc gttgccaatg atgttacaga
 321 tgagatggtc agactaaact ggctgacgga atttatgcct cttccgacca tcaagcattt tatccgtact cctgatgatg
 401 catggttact caccactgcg atccccggga aaacagcatt ccaggtatta gaagaatatc ctgattcagg tgaaaatatt
 481 gttgatgcgc tggcagtgtt cctgcgccgg ttgcattcga ttcctgtttg taattgtcct tttaacagcg atcgcgtatt
 561 tcgtctcgct caggcgcaat cacgaatgaa taacggtttg gttgatgcga gtgattttga tgacgagcgt aatggctggc
 641 ctgttgaaca agtctggaaa gaaatgcata atcttttgcc attctcaccg gattcagtcg tcactcatgg tgatttctca
 721 cttgataacc ttatttttga cgaggggaaa ttaataggtt gtattgatgt tggacgagtc ggaatcgcag accgatacca
 801 ggatcttgcc atcctatgga actgcctcgg tgagttttct ccttcattac agaaacggct ttttcaaaaa tatggtattg
 881 ataatcctga tatgaataaa ttgcagtttc atttgatgct cgatgagttt ttctaatcag aattggttaa ttggttgtaa
 961 cactggcaga gcattacgct gacttgacgg gacggcggct ttgttgaata aatcgaactt ttgctgagtt gaaggatcag
1041 atcacgcatc ttcccgacaa cgcagaccgt tccgtggcaa agcaaaagtt caaaatcacc aactggtcca cctacaacaa
1121 agctctcatc aaccgtggct ccctcacttt ctggctggat gatggggcga ttcaggcctg gtatgagtca gcaacacctt
1201 cttcacgagg cagacctcac tagttccact gagcgtcaga ccccgtagaa aagatcaaag gatcttcttg agatcctttt
1281 tttctgcgcg taatctgctg cttgcaaaca aaaaaaccac cgctaccagc ggtggtttgt ttgccggatc aagagctacc
1361 aactcttttt ccgaaggtaa ctggcttcag cagagcgcag ataccaaata ctgtccttct agtgtagccg tagttaggcc
1441 accacttcaa gaactctgta gcaccgccta catacctcgc tctgctaatc ctgttaccag tggctgctgc cagtggcgat
1521 aagtcgtgtc ttaccgggtt ggactcaaga cgatagttac cggataaggc gcagcggtcg ggctgaacgg ggggttcgtg
1601 cacacagccc agcttggagc gaacgaccta caccgaactg agatacctac agcgtgagca ttgagaaagc gccacgcttc
1681 ccgaagggag aaaggcggac aggtatccgg taagcggcag ggtcggaaca ggagagcgca cgagggagct tccaggggga
1761 aacgcctggt atctttatag tcctgtcggg tttcgccacc tctgacttga gcgtcgattt ttgtgatgct cgtcaggggg
1841 gcggagccta tggaaaaacg ccagcaacgc ggccttttta cggttcctgg ccttttgctg gccttttgct cacatgttct
1921 ttcctgcgtt atcccctgat tctgtggata accgtattac cgcctttgag tgagctgata ccgctcgccg cagccgaacg
2001 accgagcgca acgcgtgagc ccaccagctc cgtaagttcg ggtgctgtgt ggctcgtacc cgcgcattca ggcggcaggg
2081 ggtctaacgg gtctaaggcg gcgtgtacgg ccgccacagc ggctcttagc ggcccggaaa cgtcctcgaa acgacgcatg
2161 tgttcctcct ggttggtaca ggtggttggg ggtgctcggc tgtcgctggt gtttcatcat cagggctcga cgggagagcg
2241 ggggagtgtg cagttgtggg gtggcccctc agcgaaatat ctgacttgga gctcgtgtcg gaccatacac cggtgattaa
2321 tcgtggttta ttatcaagcg tgagccacgt cgccgacgaa tttgagcagc tctggctgcc gtactggtcc ctggcaagcg
2401 acgatctgct cgaggggatc taccgccaaa gccgcgcgtc ggccctaggc cgccggtaca tcgaggcgaa cccaacagcg
2481 ctggcaaacc tgctggtcgt ggacgtagac catccagacg cagcgctccg agcgctcagc gcccgggggt cccatccgct
2561 gcccaacgcg atcgtgggca atcgcgccaa cggccacgca cacgcagtgt gggcactcaa cgcccctgtt ccacgcaccg
2641 aatacgcgcg gcgtaagccg ctcgcataca tggcggcgtg cgccgaaggc cttcggcgcg ccgtcgatgg cgaccgcagt
2721 tactcaggcc tcatgaccaa aaaccccggc cacatcgcct gggaaacgga atggctccac tcagatctct acacactcag
2801 ccacatcgag gccgagctcg gcgcgaacat gccaccgccg cgctggcgtc agcagaccac gtacaaagcg gctccgacgc
2881 cgctagggcg gaattgcgca ctgttcgatt ccgtcaggtt gtgggcctat cttcccgccc tcatgcggat ctacctgccg
2961 acccggaacg tggacggact cggccgcgcg atctatgccg agtgccacgc gcgaaacgcc gaatttccgt gcaacgacgt
3041 gtgtcccgga ccgctaccgg acagcgaggt ccgcgccatc gccaacagca tttggcgttg gatcacaacc aagtcgcgca
3121 tttgggcgga cgggatcgtg gtctacgagg ccacactcag tgcgcgccat gcggccatct cgcggaaggg cgcagcagcg
3201 cgcacggcgg cgagcacagt tgcgcggcgc gcaaagtccg cgtcagccat ggaggcattg ctatgagcga cggctacagc
3281 gacggctaca gcgacggcta caactggcag ccgactgtcc gcaaaaagcg gcgcgtgacc gccgccgaag gcgctcgaat
3361 caccggacta tccgaacgcc acgtcgtccg gctcgtggcg caggaacgca gcgagtggtt cgccgagcag gctgcacgcc
3441 gcgaacgcat ccgcgcctat cacgacgacg agggccactc ttggccgcaa acggccaaac atttcgggct gcatctggac
3521 accgttaagc gactcggcta tcgggcgagg aaagagcgtg cggcagaaca ggaagcggct caaaaggccc acaacgaagc
3601 cgacaatcca ccgctgttct aacgcaattg gggagcgggt gtcgcggggg ttccgtgggg ggttccgttg caacgggtcg
3681 gacaggtaaa agtcctggta gacgctagtt ttctggtttg ggccatgcct gtctcgttgc gtgtttcgtt gcgtccgttt
3761 tgaataccag ccagacgaga cggggttcta cgaatcttgg tcgataccaa gccatttccg ctgaatatcg tggagctcac
3841 cgccagaatc ggtggttgtg gtgatgtacg tggcgaactc cgttgtagtg cttgtggtgg catccgtggc gcggccgcgg
3921 taccagatct ttaaatctag aggtgaccac aacgacgcgc ccgctttgat cggggacgtc tgcggccgac catttacggg
4001 tcttgttgtc gttggcggtc atgggccgaa catactcacc cggatcggag ggccgaggac aaggtcgaac gaggggcatg
4081 acccggtgcg ggcttcttg cactcggcat aggcgagtgc taagaataac gttggcactc gcgaccggtg agtcgtaggt
4161 cgggacggtg aggccaggcc cgtcgtcgca gcgagtggca gcgaggacaa cttgagccgt ccgtcgcggg cactgcgccc
4241 ggccagcgta agtagcgggg ttgccgtcac ccggtgaccc ccggtttcat ccccgatccg gcggcatcac gaattcaagc
4321 ttatcgatgt cgacgtagtt aactagcgta cgatcgactg ccaggcatca aataaaacga aaggctcagt cgaaagactg
4401 ggcctttcgt tttatctgtt gtttgtccgg ccatcatggc cgcggtgatc a
```
